# Lysine butyrylation modulates central carbon metabolism and photosynthesis in cyanobacteria

**DOI:** 10.1016/j.jbc.2026.113558

**Published:** 2026-09-17

**Authors:** Sihan Li, Mingkun Yang, Jian Lin, Yanyan Chen, Li Yuan, Jiao Zhan, Feng Ge

**Affiliations:** 1 Research Center for Algae and Biomanufacturing, Institute of Hydrobiology, Chinese Academy of Sciences, Wuhan, China; 2 University of Chinese Academy of Sciences, Beijing, China; 3 State Key Laboratory of Breeding Biotechnology and Sustainable Aquaculture, Institute of Hydrobiology, Chinese Academy of Sciences, Wuhan, China

**Keywords:** cyanobacteria, lysine butyrylation (Kbu), post-translational modification (PTM), pyruvate metabolism, photosynthesis, *Synechococcus* sp. strain PCC 7002 (Syn7002)

## Abstract

Cyanobacteria, evolutionarily ancient photoautotrophs, serve as key model organisms for studying diverse biological processes. Lysine butyrylation (Kbu) is an emerging post-translational modification implicated in diverse biological processes from bacteria to mammals. However, the distribution and functional significance of Kbu in cyanobacteria have yet to be elucidated. In this study, we present the first proteome-wide analysis of Kbu in the model cyanobacterium *Synechococcus* sp. strain PCC 7002 using peptide pre-fractionation, butyrylation antibody enrichment and high-accuracy mass spectrometry analysis. In total, we identified 132 high-confidence Kbu sites across 93 proteins in Syn7002. These butyrylated proteins were predominantly enriched in carbon metabolism and photosynthesis pathways, which are tightly interconnected in cyanobacteria. Immunoblotting revealed that global Kbu levels in Syn7002 varied dynamically under different growth conditions, and the change in Kbu levels of the allophycocyanin α subunit were further confirmed using parallel reaction monitoring. Among the butyrylated metabolic proteins, phosphoenolpyruvate synthase (PPSA) was found to be butyrylated at the K143 residue. By employing genetic code expansion to site-specifically incorporate Nε-butyryl-lysine at position 143 (K143bu), we demonstrated that butyrylation at this site significantly affects the enzymatic activity of PPSA *in vitro*. Consistently, the K143R mutant of PPSA in Syn7002 displayed impaired growth and reduced photosynthetic activity under high light conditions compared with the wild type, suggesting a functional role for Kbu *in vivo*. Together, our results expand the understanding of Kbu and suggest its potential involvement in regulating cyanobacterial carbon metabolism and photosynthesis.

Post-translational modifications (PTMs) of proteins involve the reversible or irreversible attachment of functional groups to amino acid side chains or to the N- and C-termini of proteins, forming covalent chemical adducts that constitute to the primary structure of the protein (1). PTMs can make significant effects on protein function, playing important roles in protein folding, signal transduction, and molecular transport (2, 3). The development of high-sensitivity mass spectrometry has facilitated the discovery of numerous novel lysine acylation modifications in recent years (4), including propionylation (5), butyrylation (5), crotonylation (6), 2-hydroxyisobutyrylation (7), β-hydroxybutyrylation (8), malonylation (9), succinylation (9), and glutamylation (10). These acylation modifications are closely associated with key biological processes (BP) such as signal transduction, metabolic stress responses, tissue injury, and transcriptional regulation (7, 11, 12, 13). Lysine butyrylation (Kbu) was first identified in 2007, when acetyltransferases p300 and CREB-binding protein (CBP) were shown to catalyze butyrylation of lysine residues in histones. Additionally, both p300 and CBP were reported to undergo self-butyrylation *in vitro* (5). In the context of spermatocyte differentiation, histone butyrylation has been shown to directly stimulate transcription and delay histone removal in spermatogenic cells (14). To date, studies of Kbu have largely focused on eukaryotic organisms, including animals, plants, and fungi, demonstrating its crucial roles in stress responses, biosynthetic processes, and human diseases (5, 15, 16, 17, 18). More recently, Kbu has been demonstrated to impact the activity of the essential metabolic enzyme Buk and regulate the auto-expression of the sporulation regulator Spo0A in the Gram-positive bacterium *Clostridium acetobutylicum* (16). Furthermore, site-specific Kbu of the transcription factor HilA in *Salmonella enterica* was shown to modulate its HilA-dependent gene expression, and attenuate serovar Typhimurium invasion of epithelial cells as well as dissemination *in vivo* (19). These findings provide accumulating evidence that Kbu also serves as an important mechanism regulating metabolism and cellular physiology in prokaryotes.

Cyanobacteria are a class of oxygen-producing photosynthetic prokaryotes and the evolutionary precursors of plant plastids (20). They contribute approximately 25% of the net primary productivity in the ocean (21), and are widely used as model organisms for studying BP including photosynthesis, circadian rhythms, and cell differentiation, while also offering broad application potential in biotechnology and synthetic biology (22, 23). The *Synechococcus* sp. strain PCC 7002 (hereafter Syn7002), a unicellular marine cyanobacterium, is characterized by its rapid growth rates as well as its tolerance to high salinity, intense light and alkaline environments. Its genome sequence is well-characterized, making it amenable to genetic manipulation and becoming one of the model species for cyanobacterial research (24). There is ongoing interest in the identification and functional characterization of PTMs in cyanobacteria. In 2014, more than 20 distinct types of PTMs—including acetylation, methylation, succinylation, and butyrylation—were systematically identified and functionally annotated in Syn7002 (25). Subsequent studies have further elucidated the functions and regulatory enzymes associated with lysine acylation modifications such as acetylation, propionylation, and crotonylation in cyanobacteria (26, 27, 28, 29). For instance, the crotonylation of the photosystem I protein PsaD in Syn7002, modulated by crotonyltransferase cGNAT2 and decrotonylase CddA, impacts the structure of PsaD, thereby affecting cell growth and the efficiency of photosynthetic electron transport (25). Additionally, acetylation at the K89 residue in NdhJ, a hydrophilic subunit of the NDH-1 complex in Syn7002, influences the activity of NdhJ and consequently affects photosynthetic electron flow and cyanobacterial growth (29). These studies have highlighted lysine acylation as critical regulatory mechanisms, particularly in the control of photosynthesis and carbon metabolism in cyanobacteria (30). Kbu is a conserved lysine acylation modification that has been identified in animals, plants, bacteria, and fungi and has been implicated in stress responses and biosynthetic processes (5, 15, 16, 17, 18), so we hypothesized that Kbu may also affect photosynthesis and carbon metabolism in cyanobacteria. However, no butyrylated proteins have been previously reported in cyanobacteria.

To obtain a comprehensive understanding of Kbu events in cyanobacteria, we studied the identities and functional roles of the butyrylated proteins in Syn7002 using antibody-based butyrylation enrichment combined with high-throughput mass spectrometry. In total, we identified 132 high-confidence Kbu sites across 93 proteins in Syn7002, representing the first extensive data set of Kbu in cyanobacteria. The identified butyrylated proteins were involved in diverse BPs, with significant enrichment in photosynthetic and metabolic pathways. The functional significance of Kbu in phosphoenolpyruvate synthase (PPSA), a key enzyme in pyruvate metabolism, was further assessed through biochemical assays and site-specific mutagenesis. These analyses indicate that butyrylation of PPSA K143 residue affects the enzymatic activity and may influence metabolism and photosynthetic performance in Syn7002. Overall, our results reveal the broad potential functions of Kbu and suggest that this modification represents a previously unrecognized mechanism regulating carbon metabolism and photosynthetic processes in cyanobacteria.

## Results

### Kbu is abundant in prokaryotes and photosynthetic organisms

To detect the relative abundance of Kbu across different prokaryotic and eukaryotic species, we used an anti-butyrylation antibody to perform Western blotting on whole cell lysates from a variety of bacteria (*Bacillus subtilis*, *Escherichia coli*, *Pseudomonas aeruginosa*, *Staphylococcus aureus* and *Salmonella typhimurium*) and photosynthetic organisms (*Synechococcus* sp. PCC 7002, *Synechocystis* sp. PCC 6803, *Anabaena* sp. PCC 7120, *Synechococcus* sp. PCC 7942, *Vacuoliviride crystalliferum*, and *Oryza sativa*) (Fig. 1, *A* and *B*). Immunoblot signals were detected in all species, indicating that Kbu is conserved across both prokaryotic and eukaryotic photosynthetic organisms. To determine whether Kbu levels are affected by external stresses, we applied multiple stress treatments to Syn7002, including high light (HL), nitrogen deprivation (-N), high salt (NaCl), and butyrate stress. Western blotting was performed on cell lysates, and overall Kbu levels were quantified through grayscale scanning. Results showed that Kbu levels were upregulated under all four stress conditions compared to the non-control (NC) group (Fig. 1*C*). Among these, the highest increase in modification levels was observed under butyrate stress, likely because short-chain fatty acids serve as substrates for their corresponding acylation modifications (4, 31). These findings indicate that Kbu is modulated by external environment. Although the global increase in Kbu levels was relatively modest (∼30%), such subtle alterations in global PTM profiles have been documented in previous studies and may underlie critical regulatory changes at the level of specific proteins or pathways (32, 33). We therefore propose that Kbu likely plays important biological roles in Syn7002.

**Figure 1:**
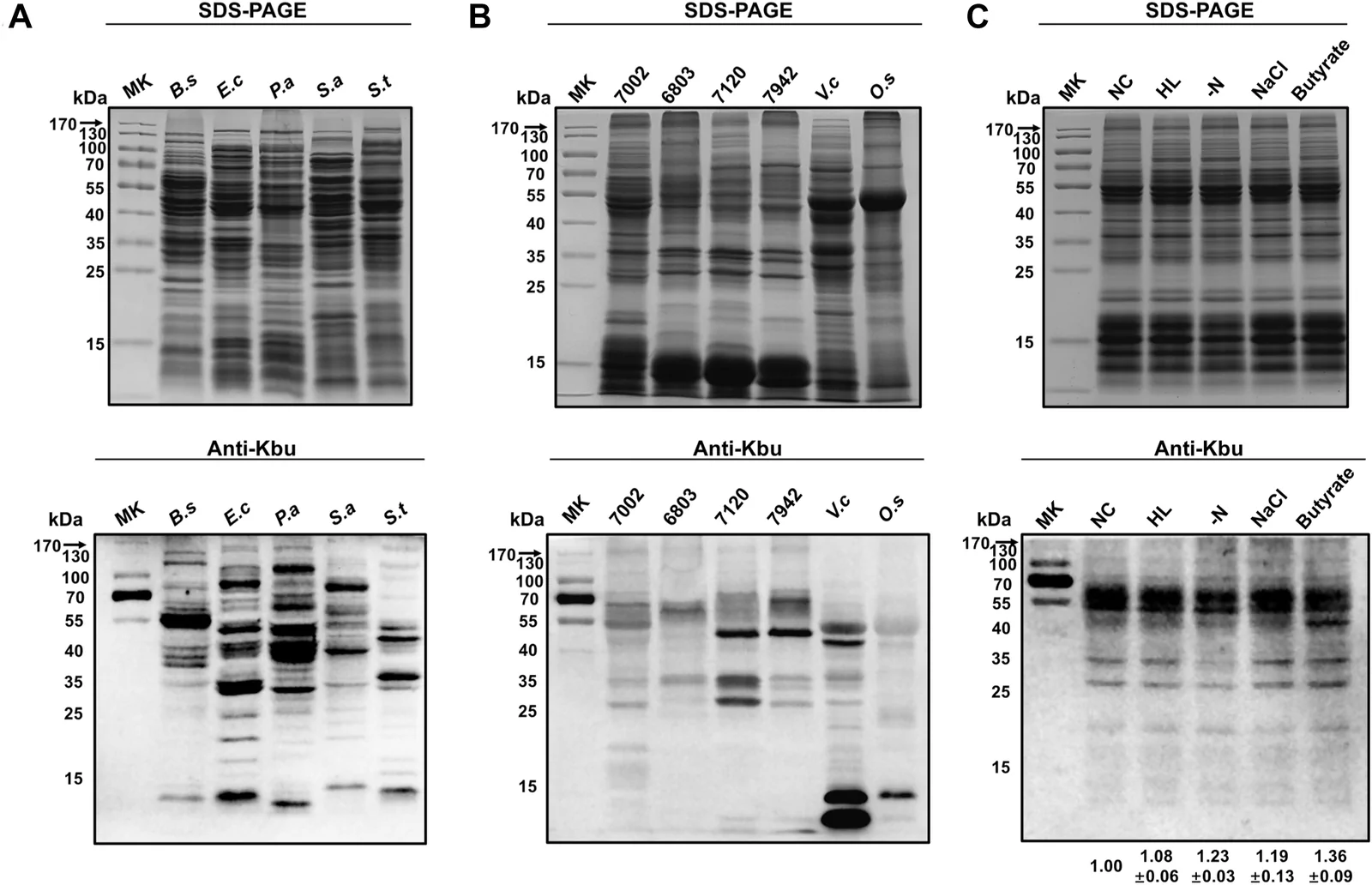
**Kbu is prevalent in prokaryotes and eukaryotes.***A*, detection of Kbu in multiple bacterial species by Western blotting. Lanes from *left* to *right*: *Bacillus subtilis*, *Escherichia coli*, *Pseudomonas aeruginosa*, *Staphylococcus aureus*, and *Salmonella typhimurium*. *B*, detection of Kbu in multiple photosynthetic organisms by Western blotting. Lanes from *left* to *right*: *Synechococcus sp.* PCC 7002; *Synechocystis sp.* PCC 6803; *Anabaena sp.* PCC 7120; *Synechococcus sp.* PCC 7942; *Vacuoliviride crystalliferum*; and *Oryza sativa*. *C*, detection of Kbu levels in Syn7002 under different conditions by Western blotting. Conditions included normal condition (NC); high light (HL); nitrogen deprivation (−N); high salt (NaCl) and butyrate stress (butyrate). *Grayscale* intensities for each lane were quantified from three independent biological replicates, and are presented as the mean ± SD. For each Western blotting shown in *panelsA* and *C*, the corresponding *white-light* image containing the molecular weight markers was used as a reference to assign the indicated molecular weights.Kbu, lysine butyrylation.

### Identification of butyrylated proteins in Syn7002

To identify butyrylated proteins and their modified sites in Syn7002, we applied a workflow involving protein extraction from multiple growth conditions, peptide pre-fractionation, butyrylation antibody enrichment and high-accuracy mass spectrometry analysis to detect Kbu (Fig. 2*A*). In total, 132 Kbu sites on 93 proteins were identified in Syn7002 (Fig. S1). The identified peptide-spectrum match (PSMs) carrying Kbu are listed in Table S3, and the detailed information on the butyrylated proteins and sites is provided in Table S4. The mass spectrometry data have been deposited in the public-access iprox database (http://www.iprox.org) with the identifier PXD072902. Among all butyrylated proteins, most possess only one Kbu site, with a maximum of five sites detected on a single protein. Then we analyzed the amino acid sequence preferences at positions -6 to +6 around butyrylated lysine residues and found that position −1 tends to be methionine, while position −2 tends to avoid leucine (Fig. S2). Secondary structure and solvent accessibility analysis of Kbu sites indicate that butyrylated lysine residues show a slightly higher tendency to occur in α-helices and a slightly lower tendency in coil structures compared with unmodified residues. However, solvent accessibility at butyrylated lysine residues shows no significant difference compared to unmodified residues (Fig. S3). The detailed secondary structure and surface accessibility of butyrylated lysine residues in Syn7002 were predicted in Table S5.

**Figure 2:**
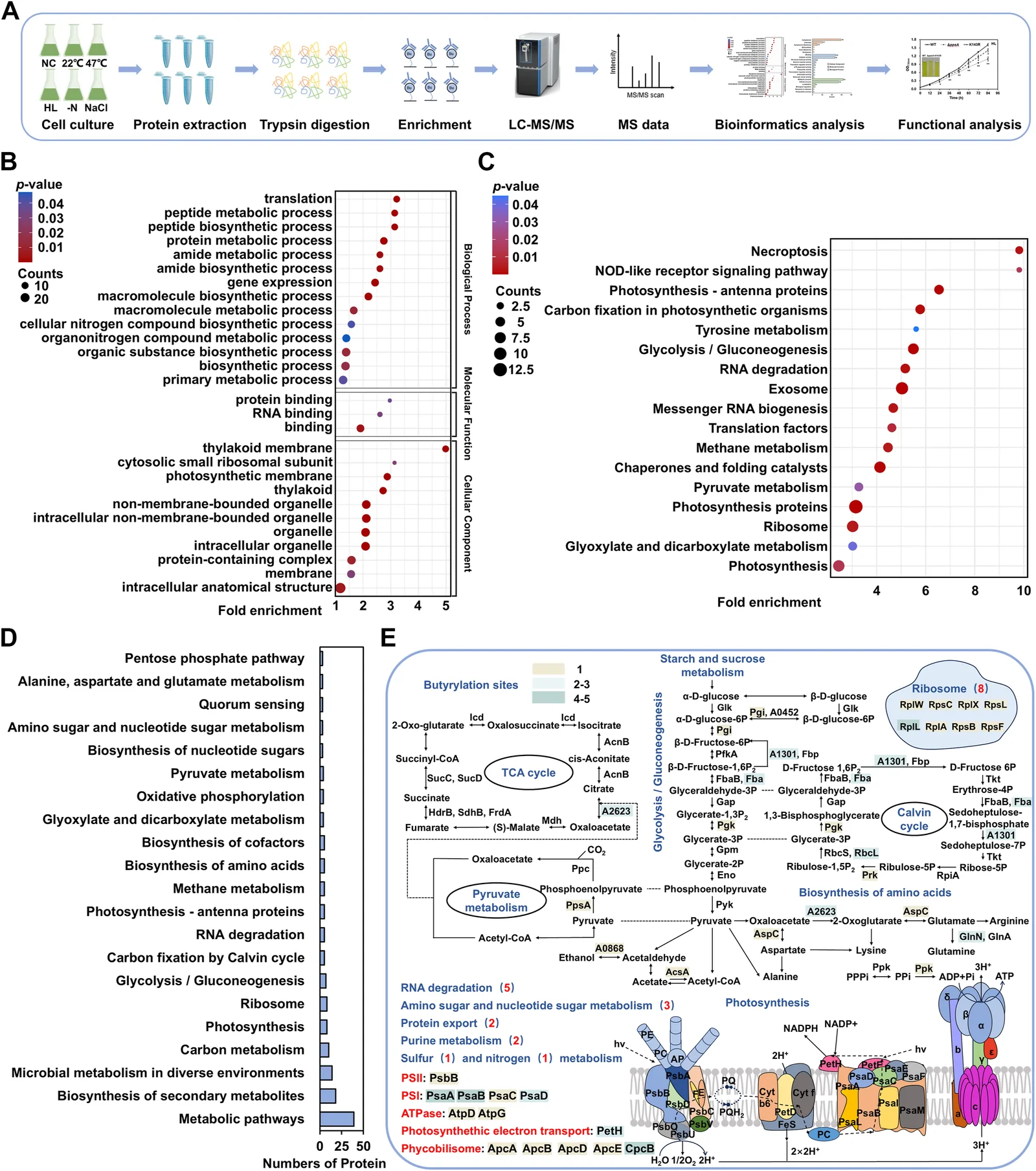
**Functional characterization of lysine butyrylated proteins in Syn7002.***A*, schematic overview of the experimental workflow used for Kbu analysis. *B*, Gene Ontology enrichment analysis of butyrylated proteins categorized by biological process, molecular function and cellular component. *C*, Kyoto Encyclopedia of Genes and Genomes pathway enrichment analysis of butyrylated proteins. *D*, illustration of Kyoto Encyclopedia of Genes and Genome pathways containing the highest numbers of lysine butyrylated proteins. *E*, mapping of butyrylated proteins within photosynthetic and metabolic pathways. Identified butyrylated proteins were highlighted in color. Kbu, lysine butyrylation.

### Functional annotation of butyrylated proteins in Syn7002

To gain deeper insights into the functions of butyrylated proteins in Syn7002, we performed Gene Ontology (GO) annotation on these proteins; followed by GO enrichment (Fig. 2*B* and Table S6) and GO classification (Fig. S4 and Table S7) analyses according to BP, molecular function (MF), and cellular component (CC). In terms of BPs, butyrylated proteins were predominantly involved in translation, biosynthetic processes, and metabolic processes. Regarding MFs, these proteins were mainly associated with binding activities. With respect to CCs, butyrylated proteins were primarily localized to organelles and various membrane-associated structures. We further performed Kyoto Encyclopedia of Genes and Genome (KEGG) pathway enrichment analysis of the butyrylated proteins, which revealed strong associations with diverse BPs (Fig. 2*C* and Table S8). In photosynthesis-related pathways, butyrylated proteins were significantly enriched in photosynthetic antenna proteins (*p* = 0.0006), carbon fixation (*p* = 0.0011), and photosynthetic proteins (*p* = 0.0001). In metabolic pathways, significant enrichment was observed in glycolysis/gluconeogenesis (*p* = 0.0002), methane metabolism (*p* = 0.0040), pyruvate metabolism (*p* = 0.0303), and glyoxylate and dicarboxylate metabolism (*p* = 0.0394). Together, these results indicate that Kbu is closely associated with multiple photosynthetic and metabolic processes in Syn7002, consistent with the functional patterns reported for other lysine acylation modifications (27, 28, 29).

### Butyrylated proteins linked to metabolism and photosynthesis

To further elucidate the photosynthetic and metabolic networks involving butyrylated proteins in Syn7002, all butyrylated proteins were mapped to KEGG pathways (Fig. 2, *D* and *E* and Table S9). As shown in Figure 2*D*, the five pathways containing the highest number of butyrylated proteins were metabolic pathways (n = 39), biosynthesis of secondary metabolites (n = 18), microbial metabolism in extreme environments (n = 14), carbon metabolism (n = 10), and photosynthesis (n = 8), similar to the KEGG pathway enrichment results. Figure 2*E* illustrates the distribution of butyrylated proteins across multiple KEGG pathways, indicating that butyrylated proteins are involved in several key pathways, including glycolysis/gluconeogenesis, photosynthesis, pyruvate metabolism, and amino acid synthesis. Notably, pyruvate metabolism, as a crucial metabolic pathway, connects to other pathways such as glycolysis/gluconeogenesis and amino acid synthesis. Moreover, phosphoenolpyruvate (PEP) participates in carbon fixation pathways in cyanobacteria, thereby linking pyruvate metabolism to photosynthesis (34). Kbu was also detected on proteins involved in pyruvate metabolism, suggesting that it may play a role in regulating carbon metabolism, and influencing photosynthetic processes in Syn7002 consequently.

### Parallel reaction monitoring -based validation and quantification of allophycocyanin α subunit K27 butyrylation under different conditions

As Syn7002 is a widely used model organism for photosynthesis research, we selected allophycocyanin α subunit (ApcA), a photosynthetic protein identified as butyrylated in our proteomic analysis, and used PRM assays to validate the modification and examine its dynamic changes under diverse growth conditions. ApcA is the α subunit of the hetero-trimeric (αβ)_3_ complex, located within the core region of the phycobilisome antenna protein. It plays a crucial role in the electron transfer process from the phycobilisome antenna to the photosystem (35). In the experiment, total protein extracts were prepared from cells grown under multiple conditions, including NC, low temperature (22 °C), high temperature (47 °C), HL, nitrogen deprivation (-N), and high salt (NaCl) conditions. Targeted PRM was subsequently employed to quantify the endogenous Kbu levels of ApcA across these conditions. The PRM data have been deposited in the public-access iprox database (http://www.iprox.org) with the identifier PXD072902. Our proteomic analysis identified a Kbu site at K27 residue of ApcA (Table S4). In the PRM analysis, a representative MS/MS spectrum of the peptide harboring the K27 butyrylation is shown in Figure 3*A*, confirming the high confidence of site localization and spectral quality. The PRM analysis consistently detected the K27-butyrylated peptide with sufficient signal intensity to enable reliable quantification, as illustrated by the extracted ion chromatograms (Figs. 3*B* and S5). Quantitative peak areas from individual biological replicates are summarized in Figure 3*C*. We then quantified the abundance of the K27-butyrylated peptide under these conditions. As shown in Figure 3*D*, the butyrylated peptide of ApcA was detected under all tested growth conditions, and the butyrylation level was significantly decreased under high salt condition compared with NC (*p* = 0.001). These results confirm the presence of Kbu on K27 residue of ApcA under diverse growth conditions in Syn7002 and indicate that the modification level can be altered under specific environmental stresses.

**Figure 3:**
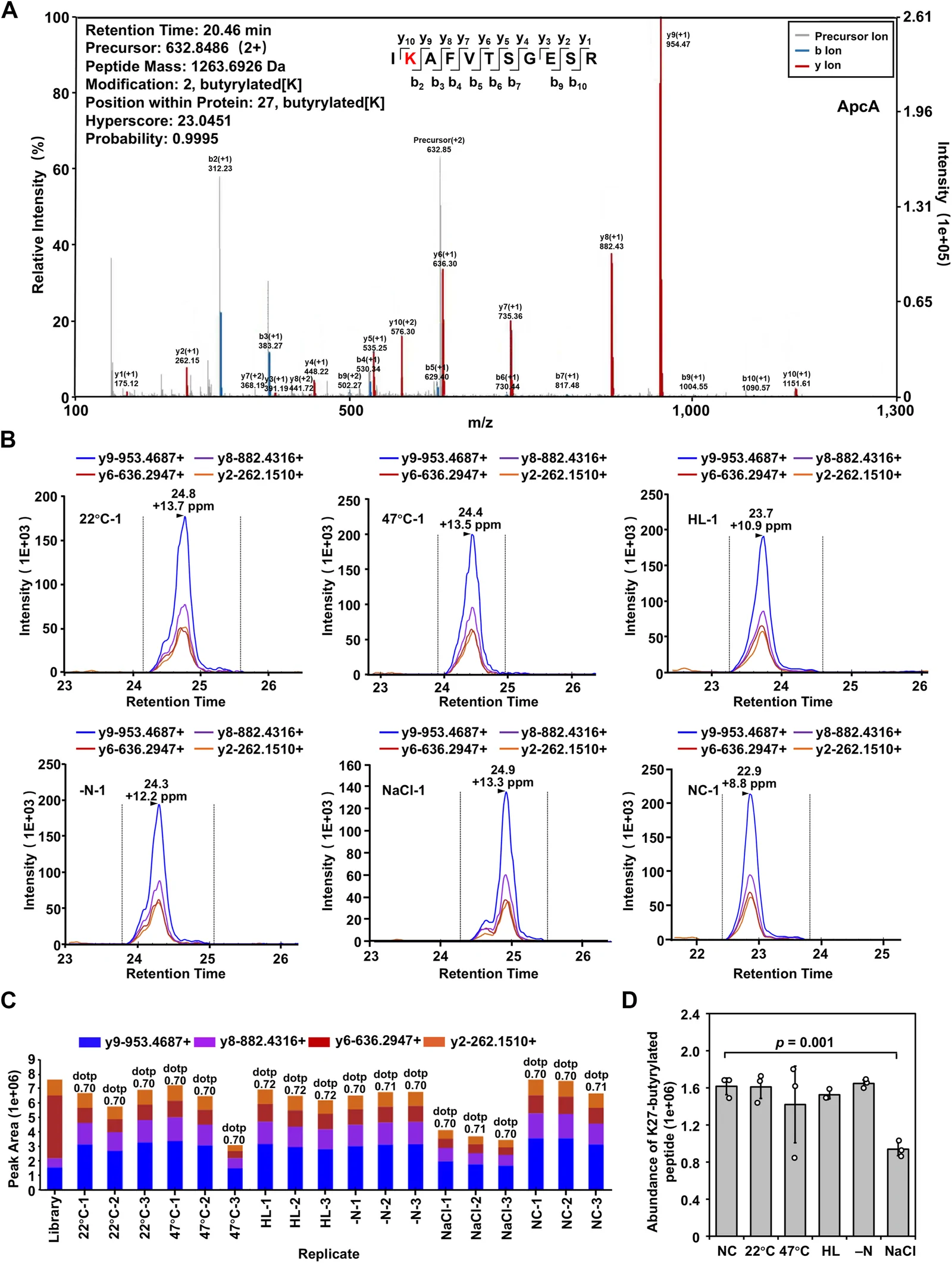
**Quantification of Kbu intensity at the K27 residue on ApcA using parallel reaction monitoring.***A*, a representative MS/MS spectrum of the K27-butyrylated peptide of ApcA. *B*, extracted ion chromatograms of the K27-butyrylated peptide of ApcA under low temperature (22 °C), high temperature (47 °C), high light (HL, 2000 μE), nitrogen deprivation (−N), high salt (NaCl), and normal conditions (NC). *C*, corresponding peak areas of the K27-butyrylated peptide quantified from parallel reaction monitoring analyses. *D*, the abundance of the K27-butyrylated peptide under the indicated conditions. Each data was obtained from three independent biological replicates. Statistical significance was determined using two-tailed Student’s *t*-tests. Kbu, lysine butyrylation.

### Butyrylation at K143 residue affects PPSA enzymatic activity *in vitro*

Kbu is dictated by intracellular butyryl-CoA availability, providing a direct link between cellular metabolic states and functions of butyrylated proteins (36). PPSA serves as one of the key enzymes in pyruvate metabolism, catalyzing the conversion from pyruvate to PEP, both of which are central metabolites in all living organisms (37). This central metabolic role provides a strong rationale for functional analysis of Kbu on PPSA (Fig. 4*A*). In this study, we identified a high-confidence Kbu site at K143 residue of PPSA in Syn7002 (accession number A0250). Structural modeling using Swiss-Model indicated that K143 residue is located within an α-helix and forms hydrogen bonds with neighboring Asp136, Glu139, and Glu147 residues (Fig. 4*B*). Therefore, we speculate that Kbu of K143 residue induces local conformational changes that disrupt hydrogen bonding interactions between K143 residue and adjacent residues by introducing a steric bulk and neutralizing the positive charge of the lysine residue, ultimately affecting the function of PPSA. Consistently, multiple sequence alignment of PPSA from various species revealed that K143 residue is highly conserved among cyanobacteria, suggesting that this residue plays a crucial role in the function of PPSA (Fig. S6).

**Figure 4:**
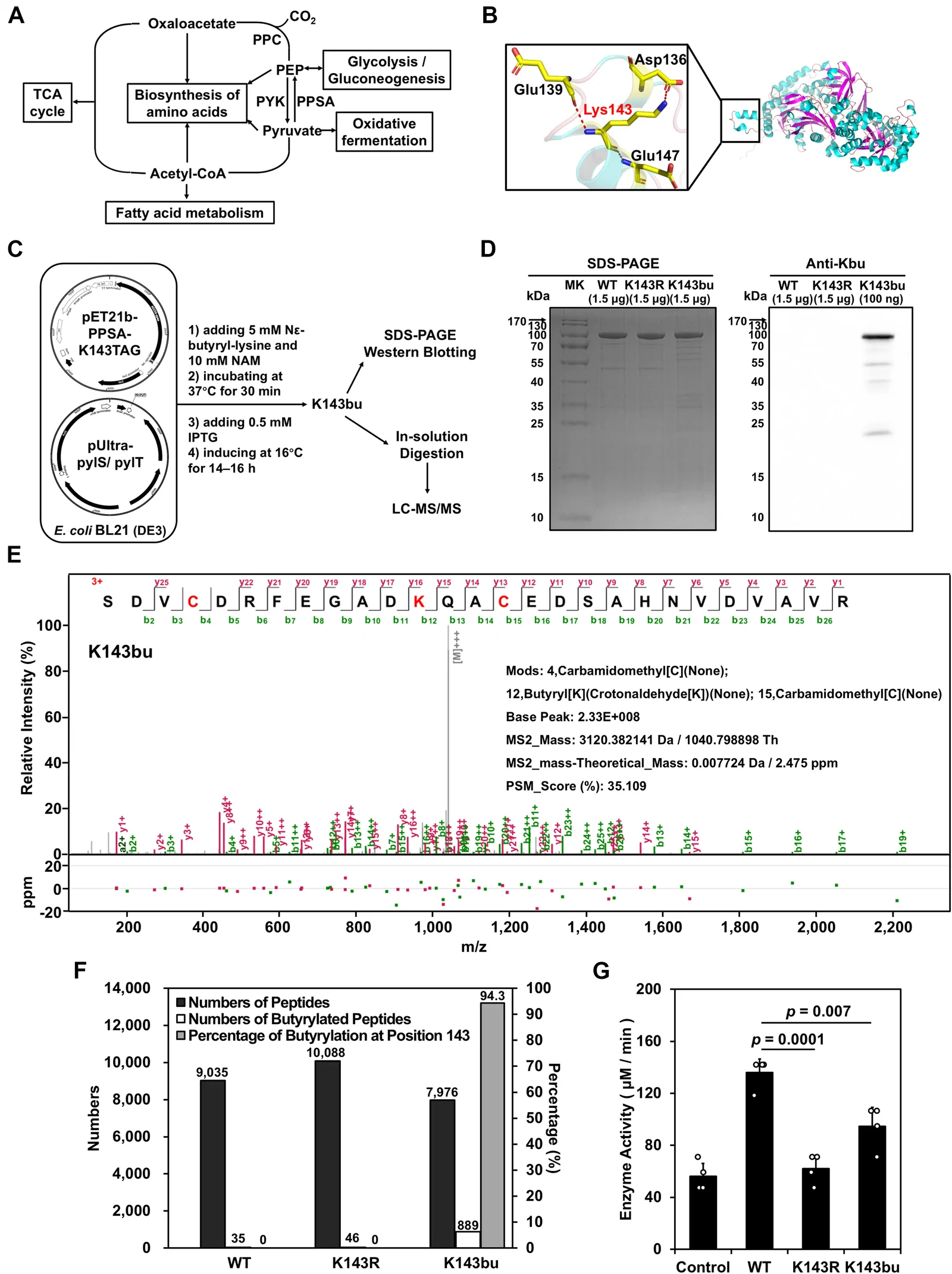
**Impact of Kbu at the K143 residue of PPSA on enzymatic activity.***A*, schematic representation of PPSA participating in cellular pyruvate metabolism. *B*, Simulation of the tertiary structure of PPSA, highlighting the K143 residue, generated using Swiss-Model. *C*, schematic illustration of the strategy used to generate and verify the K143bu variant of PPSA. *D*, SDS–PAGE and anti-Kbu Western blotting analysis of all purified PPSA variants. The amount of protein loaded in each lane is indicated above the corresponding lane. *E*, LC–MS/MS validation of K143bu variant of PPSA. Representative MS/MS spectrum of the peptide containing K143 residue is shown. *F*, summary of LC–MS/MS analysis of all purified PPSA variants, including the number of identified peptides, the number of identified butyrylated peptides, and the proportion of peptides carrying butyrylation at residue 143 among all identified peptides covering this position. *G*, enzymatic activity assay of all purified PPSA variants in the conversion of pyruvate *in vitro*. A negative control group was conducted in parallel without the supplementation of the enzyme. The initial pyruvate concentration and amount of purified protein was 5 mM and 3 μg respectively, and reactions were conducted at 37 °C for 5 min. Enzyme activity was determined based on the rate of pyruvate consumption per minute. Each data was obtained from four independent biological replicates and is presented as the mean ± SD. Statistical significance was determined using two-tailed Student’s *t*-tests. PPSA, phosphoenolpyruvate synthase.

To investigate the functional importance of the K143 residue of PPSA, K143 residue was first substituted with arginine (K143R) *in vitro*, a commonly used strategy in lysine acylation studies to prevent acylation at the target site while largely preserving the basic character of the residue (38, 39, 40). To directly assess the effect of Kbu at this site, we further employed genetic code expansion (GCE) to site-specifically incorporate Nε-butyryl-lysine at position 143 (K143bu) *in vitro* (Fig. 4*C*). The purified WT PPSA, K143R, and K143bu proteins were subsequently quantified and analyzed by SDS–PAGE, which showed that all three preparations exhibited high purity. Western blotting analysis was performed to examine Kbu of the purified PPSA variants. No butyrylation signals were observed for the WT and K143R proteins, whereas the K143bu variant exhibited a markedly stronger butyrylation signal despite being loaded at only 1/15 of the amount used for the WT and K143R samples (Fig. 4*D*). The purified proteins were subsequently subjected to in-solution digestion and analyzed by LC–MS/MS. The raw MS data have been deposited in the public-access iprox database (http://www.iprox.org) with the identifier PXD072902, and all identified PSMs are listed in Table S10. Similar numbers of identified peptides were obtained for all variants, suggesting comparable digestion and MS analysis efficiency across the samples. Quantitative analysis revealed that 35 and 46 butyrylated peptides were identified in the WT and K143R variants, respectively. However, no butyrylation was detected at residue 143 in either variant (Fig. S7). In contrast, 889 butyrylated peptides were identified in the K143bu variant, and 94.3% of the peptides containing residue 143 were found to carry Nε-butyryl-lysine at this position (Fig. 4, *E* and *F*). These findings are consistent with the Western blotting results and demonstrate the efficient and site-specific incorporation of Nε-butyryl-lysine at position 143 using the GCE system. Finally, the enzymatic activity of purified WT, K143R, and K143bu proteins were evaluated by measuring the conversion rate of pyruvate during a 5-min reaction. Compared with the WT protein, the K143R variant exhibited significantly reduced activity (*p* = 0.0001), indicating that K143 residue is important for PPSA function and suggesting that Kbu at this site may be functionally relevant to PPSA activity. Notably, the K143bu variant also exhibited significantly lower activity than the WT protein (*p* = 0.007), providing direct evidence that Kbu at K143 residue negatively regulates PPSA enzymatic activity (Fig. 4*G*). Taken together, these results indicate that K143 residue is critical for PPSA activity and that Kbu at this site exerts a negative regulatory effect on PPSA enzymatic function.

### Evidence for a role of PPSA K143 butyrylation in growth and photosynthesis in Syn7002

PPSA is one of the key enzymes at the pyruvate-phosphoenolpyruvate-oxaloacetate node, which lies at the intersection of glycolysis, the tricarboxylic acid cycle, and various other metabolic pathways (37). Consequently, alterations in PPSA-catalyzed reactions are expected to exert broad effects on global metabolic regulation. In this study, we constructed a PPSA KO strain (Δ*ppsA*) and a PPSA K143R mutant in Syn7002. In the K143R mutant, substitution of lysine 143 with arginine abolishes butyrylation at this site while largely preserving the basic character of the lysine residue. This mutation is commonly used in lysine acylation studies to mimic unacylated state (38, 39, 40). Both mutants were validated by PCR and DNA sequencing (Fig. S8). As metabolism and photosynthesis are usually inextricably linked in cyanobacteria, we compared the growth and photosynthesis activity of Δ*ppsA* and K143R mutants with that of the WT under normal light conditions (continuous illumination at an intensity of 250 μmol·photons·m^−2^s^−1^) and HL conditions (continuous illumination at an intensity of 2000 μmol·photons·m^−2^s^−1^) (Fig. 5). Under normal light conditions, the Δ*ppsA* strain exhibited growth comparable to that of the WT, whereas the K143R strain showed slightly reduced growth (*p* = 0.02 at 120 h). In contrast, under HL conditions, the K143R strain displayed a significantly lower growth rate than WT (*p* = 0.045 at 84 h), and the Δ*ppsA* strain exhibited the most pronounced growth defect (*p* = 0.002 at 84 h) (Fig. 5, *A* and *E*). Whole-cell absorption spectra were also measured under both light conditions. Under HL conditions, the Δ*ppsA* strain exhibited a slight reduction in overall pigment content compared with the other strains, whereas the ratio of the chlorophyll to phycobilin absorption peaks remained comparable among all strains and across growth conditions (Fig. S9) (41). Next, we evaluated the photosynthetic performance by measuring *Fv/Fm* (Fig. 5, *B* and *F*), ETR(II) (Fig. 5, *C* and *G*) and the photosynthetic oxygen evolution rate (Fig. 5, *D* and *H*). Under HL conditions, all three parameters were significantly reduced in both the Δ*ppsA* and K143R mutants relative to WT, whereas no significant differences were observed under normal light conditions. Consistent with these findings, Non-photochemical quenching levels were elevated in both the mutant strains compared with WT under both light conditions (Fig. S10), indicating enhanced dissipation of absorbed light energy as heat in the mutants (42). Collectively, these results suggest that the butyrylatable PPSA K143 residue is important for growth and photosynthetic performance of Syn7002 under high-light conditions, and further imply a potential role of PPSA K143 butyrylation in these processes.

**Figure 5:**
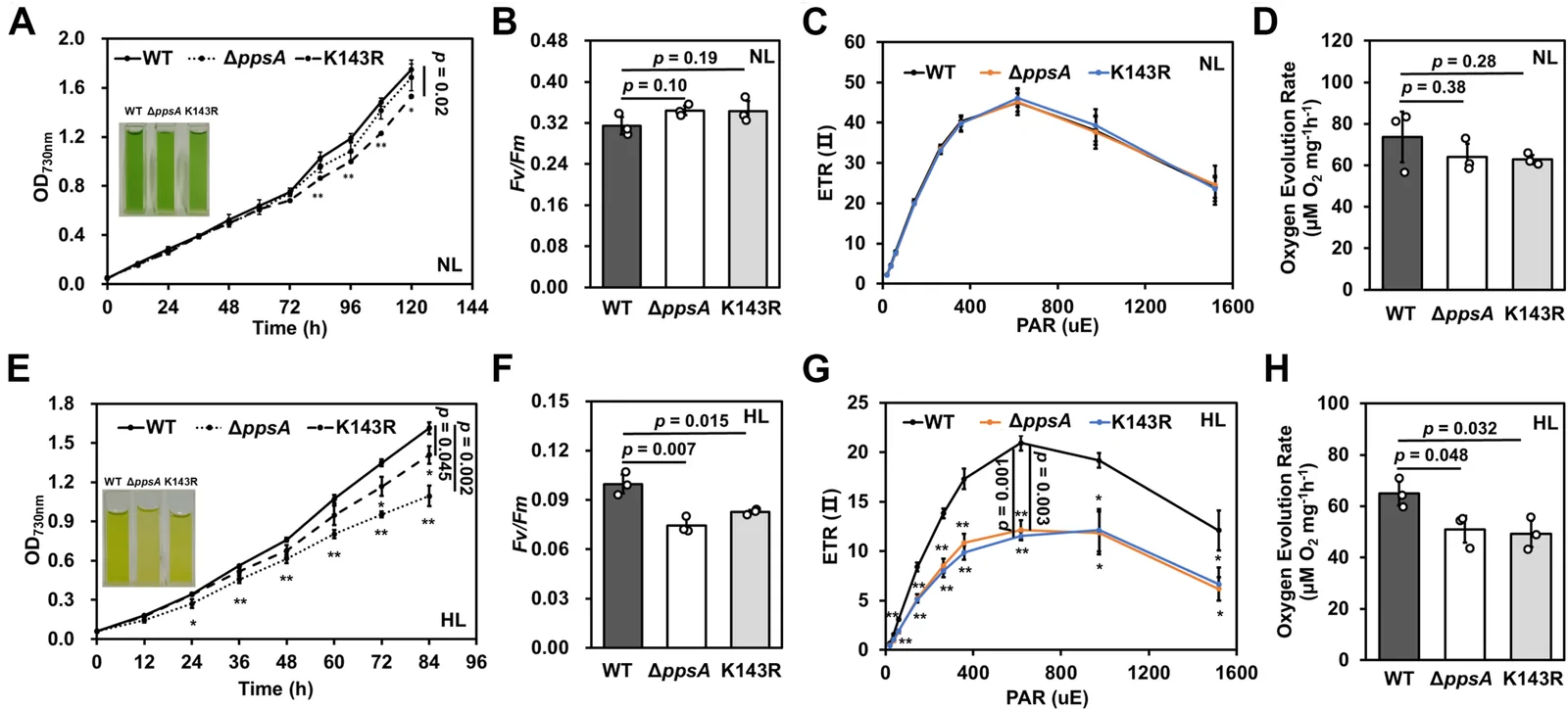
**Effect of Kbu at the K143 residue of PPSA on growth and photosynthesis in Syn 7002 under normal and HL conditions.** Growth curves, maximum PSII photochemical efficiency (*Fv/Fm*), PSII-mediated electron transport rate (ETR(II)), and photosynthetic oxygen evolution rates of WT and mutant strains (Δ*ppsA* and K143R) were measured under normal light condition (continuous illumination at an intensity of 250 μmol·photons·m^−2^s^−1^; *A–D*) and HL stress condition (continuous illumination at an intensity of 2000 μmol·photons·m^−2^s^−1^; *E–H*). Each data was obtained from at least three independent biological replicates and is presented as the mean ± SD. Statistical significance was determined using two-tailed Student’s *t*-tests. ∗ represents *p* < 0.05, while ∗∗ represents *p* < 0.01. PPSA, phosphoenolpyruvate synthase.

## Discussion

Kbu is a newly identified acylation modification reported in animals and plants. Previous studies have shown that Kbu plays important roles in spermatogenesis, adipogenesis and the onset and progression of several diseases in human beings (14, 17, 43). Kbu has also been reported to play an important role in regulating gene expression in rice, thereby influencing rice growth and development (15). In the current study, we report the first proteome-wide study of Kbu in the model cyanobacteria Syn7002. Using immunoaffinity enrichment coupled with high-precision LC-MS/MS analysis, we identified 132 Kbu sites across 93 butyrylated proteins in Syn7002. These butyrylated proteins were enriched in multiple photosynthetic and metabolic processes (Fig. 2, *B* and *C*), consistent with the functional distribution reported for other lysine acylation modification in Syn7002 (26, 27). Notably, both the number of identified butyrylated sites and proteins were markedly lower than those reported for lysine acetylation and propionylation modifications in Syn7002, which comprise 1653 acetylation sites across 802 proteins and 598 propionylation sites across 382 proteins, respectively (26, 27). Levels of acylation modifications are often modulated by the availability of their corresponding acyl-CoAs (4). Evidence have shown that in human cells, the propionyl/butyryl-CoA concentration is generally ∼10 times lower than that of acetyl-CoA, leading to the lower abundance of propionylated/butyrylated lysines (44). Consistent with this principle, supplementation with butyrate-a short-chain fatty acid precursor for butyryl-CoA biosynthesis-resulted in an approximately 30% increase in global Kbu levels in Syn7002 (Fig. 1). Given the close association between Kbu and metabolism, as well as the intimate interconnection between metabolism and photosynthesis (36), elucidating the physiological functions of butyrylated sites on metabolic enzymes provides important insights into the regulation of metabolic networks and photosynthesis in cyanobacteria.

In the present study, many proteins involved in carbon metabolism were found to be butyrylated based on MS analysis (Table S4). Among these, PPSA, catalyzing the conversion between pyruvate and PEP, was identified butyrylated at K143 residue. PPSA is one of the key regulatory enzymes in cellular pyruvate metabolism and is conserved in all three domains of life. In most organisms, the reaction strongly favors the gluconeogenic direction, towards the formation of PEP (37). However, in some thermophilic archaea, PPSA exhibits reverse catalytic activity, catalyzing the conversion of PEP to pyruvate (45). Functional evidence for PPSA in cyanobacteria remains limited. To date, only one study has reported that heterologous expression of the *ppsA* gene from Syn7002 in the engineered *Synechocystis* sp. PCC 6803 strain harboring an inserted ethylene-forming enzyme increases ethylene production, suggesting that PPSA may exert regulatory effects on metabolism (46). In this study, *in vitro* PPSA enzymatic activity was determined by measuring the rate of pyruvate consumption after addition of the purified enzyme (Fig. 4*G*). Notably, the K143bu mutant exhibited a reduced pyruvate conversion capacity compared with WT, indicating that Kbu at the K143 residue affects PPSA activity. This finding further suggests that Kbu at K143 residue may influence carbon metabolism *in vivo* through its effect on PPSA enzymatic function.

Photosynthesis and carbon metabolism are closely interconnected processes in photosynthetic organisms (47). The substrate pyruvate and the product PEP of the reaction catalyzed by PPSA are two of the 12 precursor metabolites that form the basis of all biomass on Earth (37). Pyruvate serves as a precursor for the synthesis of numerous amino acids (such as alanine, valine, and lysine) and functions as the entry point for many oxidative fermentation pathways as well. PEP is a precursor for the synthesis of aromatic amino acids and participates in carbon dioxide fixation within the C4 pathway in some C4 plants (Fig. 4*A*) (34, 37). Although cyanobacteria lack a typical C4 photosynthetic pathway, they can nevertheless utilize PEP for CO_2_ fixation, which accounts for approximately 20% of the total carbon fixed during steady-state photosynthesis (48). Consistent with this, the upregulation of *pepc* gene (encoding phosphoenolpyruvate carboxylase, an enzyme involved in the fixation of CO_2_ in diverse photosynthetic organisms) in *Anabaena* sp. PCC 7120 was reported to enhance the net photosynthesis (49). Therefore, we propose that loss of PPSA disrupts one route of pyruvate metabolism, thereby perturbing overall carbon metabolism and ultimately leading to the reduced growth and photosynthetic performances observed in Δ*ppsA* compared with WT under high light conditions (Fig. 5). Similar to the Δ*ppsA* mutant, the K143R mutant also exhibited a comparable phenotype, including the slower growth under both light conditions and the reduced photosynthetic performances under HL conditions. Given that substitution of lysine 143 with arginine abolishes butyrylation at this site, these phenotypic changes suggest that disruption of the butyrylatable K143 residue compromises PPSA function *in vivo*. Together with the *in vitro* evidence showing that Kbu at K143 residue affects PPSA activity, these results suggest that PPSA butyrylation at K143 residue may influence carbon metabolism, thereby affecting growth and photosynthesis in Syn7002.

GCE has emerged as an alternative strategy for investigating the functions of PTMs. This approach exploits the cellular translation machinery to site-specifically incorporate unnatural amino acids (UAAs) carrying PTMs or PTM mimics into proteins during translation, thereby enabling direct functional analysis of defined modifications at specific residues (50). A key requirement for this strategy is the availability of an orthogonal aminoacyl-tRNA synthetase/tRNA pair capable of specifically recognizing the desired UAA and decoding the reassigned codon (51). At present, the most widely used orthogonal systems for UAA incorporation are derived from the pyrrolysyl-tRNA synthetase/tRNA^Pyl^ from *Methanosarcina* species or tyrosyl-tRNA synthetase/tRNA^Tyr^ from *Methanococcus jannaschii* (52). In this study, we employed the (pyrrolysyl-tRNA synthetase)/tRNA^Pyl^ system to heterologously express the PPSA protein of Syn7002 in *E. coli* with Nε-butyryl-lysine site-specifically incorporated at position 143, providing a preliminary *in vitro* attempt to directly investigate the functional relevance of PPSA butyrylation. To date, although GCE has been applied predominantly in bacteria and mammalian systems, its use has gradually expanded to other organisms, including worms, fruit flies and zebrafish (53). In cyanobacteria, however, the application of GCE remains limited. In 2017, GCE was implemented in *Synechococcus elongatus* PCC 7942, where suppression of the UAG stop codon using an archaeal orthogonal translation system enabled the incorporation of UAAs into green fluorescent protein (54). Later, the same group exploited the GCE system to incorporate N-propargyl-l-lysine (PrK) into the membrane protein cytochrome bd in *S*. *elongatus* PCC 7942 to distinguish its cellular localization (55). Nevertheless, studies utilizing GCE to investigate the function of PTMs in cyanobacteria remain unexplored. Notably, GCE has already been used in photosynthetic bacteria to introduce UAAs into photosynthetic reaction center proteins, highlighting its potential for probing photosynthesis-related mechanisms (56). Therefore, the development of a more versatile GCE system in cyanobacteria could provide a powerful platform for directly investigating the functions of PTMs *in vivo*, including Kbu, in photosynthesis and metabolic regulation.

PTMs are often dynamically regulated. Immunoblot analysis using an anti-Kbu antibody revealed that global Kbu levels in Syn7002 were upregulated under all four stress conditions compared with the normal condition (Fig. 1). The stress-induced changes in Kbu levels of the ApcA were further verified by PRM analysis (Fig. 3). The dynamic nature of Kbu suggests a potential role of Kbu in stress acclimation of cyanobacteria. We also evaluated GluC as an alternative digestion strategy to assess the potential influence of protease selection on Kbu profiling. However, reliable quantification of Kbu-modified peptides could not be achieved using the GluC-based workflow, suggesting that the abundance of detectable Kbu-modified peptides may be relatively low under these conditions. This technical limitation should therefore be considered when interpreting the quantitative Kbu measurements reported in this study. To better understand the regulatory roles of Kbu in metabolism and photosynthesis, the identification of enzymes responsible for addition and removal of Kbu is necessary. The Kbu “writers” and “erasers” in animals have been intensively reported. For example, butyrylation in histones can be catalyzed by acetyltransferases p300 and CBP (5). Additionally, HBO1 is found to be a multifunctional histone acyltransferase that catalyzes not only histone butyrylation but also acetylation, propionylation, and crotonylation both *in vivo* and *in vitro*, acting in a JADE or BRPF family scaffold protein-dependent manner (57). Conversely, sirtuins are nicotinamide adenine dinucleotide-dependent human histone deacetylases catalyzing various deacylation reactions. Among the sirtuins, SIRT7 exhibits potent debutyrylation activity towards butyrylated nucleosomal histone H3 at K36 and K37 residues (58). Substantial evidence supports the idea that Kbu is similar to acetylation and is regulated by the same set of enzymes (59). In Syn7002, cGNAT2 has been shown to catalyze lysine acetylation both *in vivo* and *in vitro* (29), while CddA possesses both deacetylase and decarboxylase activities (27). However, whether these reported enzymes are also responsible for addition or removal of Kbu, or whether additional Kbu “writers” or “erasers” exist in cyanobacteria, still remains unclear and warrants further investigation.

Among the 132 butyrylated sites across 93 proteins identified in this study, the vast majority were also modified by other acylation modifications (Fig. S11), which were listed in Table S11 (26, 27, 38, 60). Numerous studies have demonstrated that distinct PTMs can be coordinately regulated in BPs. For example, in multiple cells of *Homo sapiens*, the acetylated residues in Smad7 can also be modified by ubiquitination, and the occurrence of acetylation can protect Smad7 from being ubiquitinated and degraded by the ubiquitin ligase Smurf1 (61). Emerging evidence also highlights crosstalk between Kbu and other PTMs. During mouse sperm cell differentiation, histone butyrylation not only directly promotes transcription but also competes with acetylation at histone H4 K5, preventing the binding of the bromodomain-containing protein Brdt and thereby mediating stage-specific gene expression programs (14). Similarly, a recent study on *Clostridium butyricum* reported that acetylation and butyrylation exert similar influences on the upregulation of pathways involved in genetic information processing and metabolism during two growth phases (4–12 h and 12–22 h), suggesting functional synergy between these modifications during bacterial growth (62). Therefore, it is expected that a complex interplay and cross-talk exist between protein butyrylation and other PTMs, and their corresponding influences on protein functions and photosynthesis awaits further exploration in Syn7002.

In conclusion, we report Kbu in cyanobacteria for the first time and reveal their functional relevance in cyanobacterial carbon metabolism and photosynthesis, contributing to our understanding of the complex regulatory mechanisms in cyanobacteria.

## Experimental procedures

### Cyanobacterial growth conditions

Syn7002 cells were cultured in liquid A+ medium, supplemented with 1 g/L NaNO_3_ as the nitrogen source (63). The cultivation was performed at 37 °C under continuous illumination at an intensity of 250 μmol·photons·m^−2^s^−1^, with an air supply containing 1% (v/v) CO_2_. For growth curve measurements, cells in the exponential phase (OD_730nm_ = 0.8–1.0) were transferred to fresh A+ medium until OD_730nm_ reached 0.05, after which OD_730nm_ was measured every 12 h. For nutritional stress treatments, cells in the exponential phase were centrifuged at 1800*g* for 10 min and subsequently washed three times with the corresponding stress medium. Nitrogen-deprivation, high salt, and butyrate stress media were derived from A+ medium without the addition of NaNO_3_, with the addition of 2.5 M NaCl or 5 mM sodium butyrate, respectively. HL stress was applied under continuous illumination at an intensity of 2000 μmol·photons·m^−2^s^−1^. Low temperature stress and high temperature stress were applied at 22 °C and 47 °C, respectively. All stress treatments were conducted for 2 h. After stress treatment, cells were collected by centrifugation at 1800 g for 10 min and washed three times with PBS buffer.

### Protein extraction and concentration measurement

The collected cells were resuspended in PBS buffer, and 100 mM PMSF was added to a final concentration of 1%(v/v) to inhibit protease activity. The mixture was placed on ice, and the cells were sonicated using a JY92-IIN ultrasonicator (Ningbo Scientz Biotechnology Co, Ltd). Sonication was performed for 30 min at 28% power, with a 3-s on/3-s off pulse cycle, and the alarm temperature was set to 25 °C. After sonication, the mixture was centrifuged at 13,000*g* for 15 min. The supernatant was collected, and the protein concentration was quantified using a BCA assay kit (Beyotime Institute of Biotechnology catalog no.: P0011). For Western blotting, 5 × SDS loading buffer (25% (v/v) 1 M Tris-HCl [pH 6.8], 80 mg/ml SDS, 1 mg/ml bromphenolblau, 40% (v/v) glycerin, 5% (v/v) β-mercaptoethanol) was added to a specified amount of protein, and the mixture was boiled for 5 min before being stored at −80 °C.

### Western blotting

Proteins of different sizes were separated by 12% (w/v) SDS-PAGE. Following electrophoresis, the gels were stained with Coomassie blue R250 solution (1 g/L Coomassie blue R250, 10% (v/v) acetic acid, 25% (v/v) isopropanol) and subsequently destained in a solution containing 10% (v/v) acetic acid and 5% (v/v) methanol. For Western blotting, the separated proteins were transferred to a polyvinylidene difluoride membrane. The membrane was incubated in blocking solution at room temperature for 2 h. The blocking solution consisted of TBST (25 mM Tris-HCl [pH 8.0], 150 mM NaCl, 0.1% (v/v) Tween 20) containing 2.5% (w/v) bovine serum albumin. The membrane was subsequently incubated with the primary antibody overnight at 4 °C. After incubation, the membrane was washed three times with TBST for 15 min each. The membrane was then incubated with the secondary antibody at room temperature for 1 h, followed by three 15-min washes with TBST. The chemiluminescence was enhanced using an immunoblotting assay kit (Advansta, Menlo Park, CA, catalog no.: K-12045-D50) and detected using a scanner (ImageQuant, GE Healthcare). Quantification analysis was carried out using ImageJ software (64). In this experiment, an anti-butyrylated lysine antibody (PTM BIO, catalog no.: PTM-337RM) was used as the primary antibody at a dilution of 1:1000, and a goat anti-rabbit antibody conjugated with HRP (Jackson ImmunoResearch, catalog no.: 111-035-003) was used as the secondary antibody at a dilution of 1:5000.

### In-solution trypsin digestion and peptide enrichment

The protein extract was reduced with 25 mM DTT at 37 °C for 45 min, and alkylated with 50 mM iodoacetamide at room temperature in the dark for 30 min. Then, 1% trypsin (Promega, Madison, WI, catalog no.: V5111) was added, and the mixture was incubated at 37 °C for at least 20 h for enzymatic digestion. Self-packed C18 columns were prepared by packing 500 mg of C18 material (40 μm particle size, 60 Å pore size; Agilent Technologies) into 15 ml AGT Cleanert SPE columns (∼3 ml bed volume). Columns were then washed five times with 10 ml of 100% acetonitrile (ACN) and equilibrated with the same volume of 0.1% TFA. After Digestion, peptides were loaded onto three self-packed C18 columns, and were sequentially washed five times with 2.0 ml of 0.1% TFA for desalting. Peptides were eluted with 2.0 ml of 0.1% TFA containing increasing concentrations of ACN (10%, 15%, 20%, 25%, 30%, 35%, 40%, 50%, 60%, and 100%) (65). After a drying process with a vacuum centrifuge, the enzymatically cleaved peptide fragments were resuspended in NETN buffer. The NETN buffer was formulated with 0.5% NP-40, 1 mM EDTA, 50 mM Tris-HCl and 100 mM NaCl, at pH 8.0.Agarose magnetic beads (Thermo Fisher Scientific, catalog no.: 88,803) conjugated with lysine butyrylated pan-antibodies (PTM BIO, catalog no.:PTM-337RM) were added, and the mixture was incubated overnight at 4 °C with gentle rotation. The magnetic beads were sequentially washed three times with NETN buffer, twice with ETN buffer (1 mM EDTA, 50 mM Tris-HCl, 100 mM NaCl, pH 8.0), and three times with H_2_O. After being eluted with 0.1% TFA, the peptide fragments were desalted using the self-packed C18 STAGE column, and subjected to mass spectrometry analysis.

### LC-MS/MS analysis

The enriched peptide fragments were redissolved in mobile phase A (0.1% formic acid) and separated using a Vanquish Neo HPLC system (Thermo Fisher Scientific). Mobile phase B consisted of 0.1% formic acid and 80% ACN. The flow rate was maintained at 400 nl/min for 22.6 min. The elution gradient was as follows: 0 to 0.5 min, 4% solvent B;0.5 to 0.6 min, 4%–8% solvent B; 0.6 to 13.6 min, 8%–22.5% solvent B; 13.6 to 20.5 min, 22.5%–35% solvent B; 20.5 to 20.9 min, 35%–55% solvent B; 20.9 to 21.4 min, 55%–99% solvent B; 21.4 to 22.6 min, 99% solvent B. The separated peptides were ionized by electrospray at a voltage of 1.9 kV and detected using an Orbitrap Astral mass spectrometer (Thermo Fisher Scientific) in data-independent acquisition (DIA) model with 300 DIA windows. Precursor ions were analyzed at the Orbitrap detector, and the fragment ions were analyzed at the Astral detector. The full MS scan resolution was set to 240,000 over a scan range of 380 to 980 m/z. Precursor ions were fragmented using high-energy collision dissociation with a normalized collision energy of 25%. The MS/MS scan was performed with the first mass fixed at 150.0 m/z and a resolution of 80,000. The automatic gain control (AGC) target was set to 500%, with a maximum injection time of 3 ms.

### Data search and analysis

All raw data were analyzed using FragPipe (version 22.0) by direct database searching of the DIA data using the MSFragger-DIA (v4.1) (66). The *Synechococcus* protein database was obtained from the available Cyanobase database (http://genome.annotation.jp/cyanobase/SYNPCC7002). A reversed decoy database was appended to estimate the false discovery rate (FDR), and a common contaminant protein database was included. A maximum of two missed cleavage sites was allowed. The mass error tolerances for precursor and fragment ions were set to 10 ppm and 20 ppm, respectively. Carbamidomethylation was specified as a fixed modification, whereas deamidation (Asn/Gln), oxidation (Met), butyrylation (Lys), and acetylation (protein N-term) were set as variable modifications. The minimum peptide length was set to seven amino acids. The maximum FDR for PSM identification, proteins, and modification sites was set to 1%. The resulting MSFragger-DIA identifications were further processed using MSBooster, which employed DIA-NN (version 1.8.2) for deep-learning-based prediction of fragment spectra, retention times, and ion mobility values (67). The predicted features were incorporated into PSM scoring and validation using Percolator. The validated identifications were subsequently used to generate an experimental spectral library using the SpecLibGen/easyPQP module implemented in FragPipe. Finally, modification sites bearing localization probability exceeding 0.75 were classified as Class I sites. Identified peptides carrying C-terminal butyrylation were discarded prior to bioinformatics analysis to enhance data quality.

### DIA and PRM experiments

Peptides generated from samples collected under different growth conditions were separated using an online nano-flow EASY-nLC 1200 system (Thermo Fisher Scientific) equipped with a 15 cm × 75 μm reversed-phase C18 analytical column. Peptide separation was performed using solvent B (0.1% formic acid in 80% ACN, v/v) with a linear gradient over 125 min as follows: 3 to 6% solvent B from 0 to 1 min, 6 to 17% from 1 to 61 min, 17 to 23% from 61 to 86 min, 23 to 32% from 86 to 105 min, 32 to 38% from 105 to 115 min, followed by a rapid increase to 95% solvent B from 115 to 116 min and maintained at this concentration for an additional 9 min. Eluting peptides were analyzed using a Q Exactive HFX mass spectrometer (Thermo Fisher Scientific) operated in DIA mode. Full MS scans were acquired over a mass range of 300–1800 m/z at a resolution of 60,000 using 80 DIA windows. The AGC target was set to 3 × 10^6^ with a maximum injection time of 20 ms. Following each survey scan, precursor ions were isolated using a 6 m/z window and fragmented by higher-energy collisional dissociation with a normalized collision energy of 28%, and fragment ions were detected at a resolution of 30,000. For MS/MS scans, the AGC target was set to 1 × 10^6^ with the maximum injection time set to auto.

DIA raw files were processed using FragPipe (version 22.0) by direct database searching of the DIA data using the MSFragger-DIA (version 4.1) against the *Synechococcus* protein database (66). Trypsin was specified as the digestion enzyme, allowing up to two missed cleavage sites. Carbamidomethylation was specified as a fixed modification, whereas deamidation (Asn/Gln), oxidation (Met) and butyrylation (Lys) were set as variable modifications. The precursor and fragment mass tolerances were set to 10 ppm and 20 ppm, respectively. Peptide and protein identifications were filtered to an FDR of 1%. The resulting MSFragger-DIA identifications were further processed using MSBooster, which employed DIA-NN (version 1.8.2) for deep-learning-based prediction of fragment spectra, retention times, and ion mobility values (67). The predicted features were incorporated into PSM scoring and validation using Percolator. The validated identifications were subsequently used to generate an experimental spectral library using the SpecLibGen/easyPQP module implemented in FragPipe. Finally, the identification result was subsequently imported into Skyline (https://skyline.ms/) software to construct a spectral library. Based on the spectral library results, the transition lists of the target tryptic peptides were generated (Table S1). For PRM experiments, peptide samples were analyzed on the same Q Exactive HFX mass spectrometer under identical chromatographic conditions, with data acquired in PRM mode. The acquired PRM datasets were analyzed using Skyline as previously described, in which chromatographic peak areas were automatically extracted from Savitzky– Golay–smoothed traces (29). To improve the confidence of peptide identification and quantification, at least three transitions per peptide were monitored to provide sufficient fragment ion evidence and enhance assay specificity (68). Peak integration boundaries were manually inspected and adjusted to ensure accurate peptide identification and quantification, following established criteria (69, 70, 71). For butyrylation stoichiometry analysis, the abundance of butyrylated peptides was calculated from their corresponding peak area. Fold changes between experimental groups were calculated based on mean values obtained from biological replicates.

### Bioinformatics analysis

All identified butyrylated proteins were annotated for GO terms using InterProScan (version 5.5) (72), and classified into BP, CC, and MF categories using the WEGO bioinformatics resources (73). Enrichment analysis of butyrylated proteins was performed with the Cytoscape plugin BiNGO v2.44 (74). The motifs of the six amino acids upstream and downstream of butyrylated lysines were analyzed using PLogo (75). The secondary structure of butyrylated lysines was predicted using NetSurfP (76). The Mapper tool on the KEGG website was used to map the butyrylated proteins to specific KEGG pathways (77). Protein structures were predicted using Swiss-Model (78), and visualized using PyMOL (v. 3.1.6) (79). For protein sequence conservation analysis, protein sequences from corresponding species were downloaded from the National Center for Biotechnology Information (NCBI) website, and sequence alignment was visualized using CLC Sequence Viewer 8.

### Protein expression and purification

The *ppsA* gene was amplified from the Syn7002 genome and ligated into the pET-21b vector (miaolingbio, catalog no.: P0016). The plasmid for K143R mutant was subsequently generated by introducing base pair substitution using overlapping PCR. The plasmid was then transformed into *E. coli* BL21 (DE3), and transformants were selected on LB agar plates containing 100 μg/ml ampicillin. A single transformant was inoculated into fresh LB medium containing the same antibiotics and cultured until the OD_600nm_ reached 0.4 to 0.6. IPTG was then added to a final concentration of 0.5 mM. After induction overnight at 16 °C, cells were collected, placed on ice, and sonicated using a JY92-IIN ultrasonicator. Sonication was performed for 30 min, with a 3-s on/3-s off pulse cycle at 26% power. The alarm temperature was set to 25 °C. After sonication, the mixture was centrifuged at 13,000 g for 15 min, and the supernatant was transferred to a Ni-NTA agarose column (Qiagen, catalog no.: 30,230). The column was sequentially washed with 5 ml of washing buffer containing 20 mM, 30 mM, or 60 mM imidazole (20 mM Tris-HCl, 500 mM NaCl, pH 8.0), respectively. The target protein was subsequently eluted with 5 ml of elution buffer (20 mM Tris-HCl, 250 mM imidazole, 500 mM NaCl, pH 8.0). The purified protein was then loaded onto a 10 kDa MWCO concentrator (Millipore, catalog no.: UFC9010) with PBS buffer. Centrifugation was performed at 4250*g* for 1 h at 4 °C, and the supernatant was collected. This procedure was repeated twice. The purified protein was validated by SDS-PAGE and quantified using a BCA assay kit (80). All primers used are listed in Table S2, and all overexpression plasmids were verified by PCR and DNA sequencing.

### Incorporating Nε-butyryl-lysine into PPSA using genetic code expansion system

The incorporation of Nε-butyryl-lysine at position 143 of PPSA was achieved using a GCE system in *E. coli*, as previously described (81, 82). The *ppsA* gene was amplified from the Syn7002 genome and ligated into the pET-21b vector. The pET21b-PPSA-K143TAG plasmid was subsequently generated by introducing an amber stop codon (TAG) using overlapping PCR. The pUltra-pylS/pylT plasmid was commercially synthesized and constructed by Jiangsu Genecefe Biotechnology Co, Ltd according to our design. Specifically, the WT *Methanosarcina mazei pylS* sequence followed by the *E. coli rrnB* terminator, together with a DNA fragment containing *Methanosarcina mazei i pylT* sequence flanked by the *E. coli proK* promoter and terminator, was commercially synthesized and subsequently assembled into the pUltra-CNF vector backbone (miaolingbio, catalog no.: P56063). The resulting plasmid was designed to express *pylS* and *pylT* under the control of tac promoter and *proK* promoter, respectively. The complete sequence of the pUltra-pylS/pylT plasmid is provided in Supplementary Methods, with major genetic elements annotated.

The pET21b-PPSA-K143TAG and pUltra-pylS/pylT plasmids were co-transformed into *E. coli* BL21 (DE3), and transformants were selected on LB agar plates containing 100 μg/ml ampicillin and 100 μg/ml spectinomycin. A single transformant was inoculated into fresh LB medium containing the same antibiotics and cultured until the OD_600nm_ reached approximately 0.4. Nε-butyryl-lysine (MCE, catalog no.: HY-W048217) was added to a final concentration of 5 mM. Optionally nicotinamide was simultaneously added at this point to a final concentration of 10 mM to inhibit potential de-butyrylation of the incorporated modified amino acid. After incubation at 37 °C for 30 min, IPTG was added to a final concentration of 0.5 mM, and protein expression was induced at 16 °C for 14 to 16 h. The recombinant protein was purified as described in the Protein Expression and Purification section, and subsequently verified by Western blotting and LC-MS/MS. All primers used are listed in Table S2, and plasmids were verified by PCR and DNA sequencing.

### Construction of mutants in Syn7002

Mutant strains were constructed using homologous recombination, as previously described (80). The upstream and downstream homologous fragments of the *ppsA* gene were amplified by PCR from the Syn7002 genome. The kanamycin resistance cassette was amplified from the pRL446 plasmid (83). For construction of the Δ*ppsA* strain, the *ppsA* coding region was replaced with the kanamycin resistance cassette. For construction of the K143R strain, the *ppsA* gene carrying the K143R substitution was amplified using primers containing the nucleotide substitutions, and the kanamycin resistance cassette was fused downstream of the mutated *ppsA* fragment. The resulting fragments were assembled by fusion PCR and ligated into the pMD-18T vector (Takara, catalog no.: 6011) at 4 °C overnight. The plasmids were first transformed into *E. coli* DH5α, and transformants were selected on LB agar plates containing 50 μg/ml kanamycin. Plasmids extracted from the confirmed transformants were subsequently introduced into Syn7002 cells in the exponential phase. Briefly, 1 ml Syn7002 cells in the exponential growth phase were harvested and resuspended in 200 μl of fresh A+ medium. Plasmid DNA (10 μl) was then added, and the mixture was incubated for 12 h at 37 °C, with gentle mixing every 2 h. The suspension was spread onto mixed-fiber ester membranes (Xinyapaper, catalog no.: 100,160) and incubated on antibiotic-free A+ solid medium for 18 h. The membranes were subsequently transferred to A+ solid medium containing 25 μg/ml kanamycin for transformants selection (80, 84). All primers used are listed in Table S2, and all pMD-18T construct as well as Syn7002 mutants were verified by PCR and DNA sequencing.

### Measurement of chlorophyll fluorescence parameters

Chlorophyll fluorescence parameters were measured using a Dual-PAM-100 instrument (Heinz Walz GmbH, Effeltrich), including the maximum PSII photosynthetic efficiency (*Fv/Fm*), PSII-mediated electron transport rate (II), and non-photochemical quenching. Cells were collected during the exponential phase, and the OD_730nm_ was adjusted to 1.0. Each measurement was conducted after a 15-min dark adaption. The methods for measuring and calculating these parameters were as described in previous studies (29, 85).

### Measurement of photosynthetic oxygen evolution rate

An oxygen electrode system (Hansatech Instruments Limited) was used to measure the photosynthetic oxygen evolution rate. Cells were collected during the exponential phase, and the OD_730nm_ was adjusted to 0.8 before adding NaHCO_3_ to a final concentration of 10 mM. Measurements were conducted in a 37 °C water bath, assessing the oxygen consumption rate under dark conditions and the oxygen evolution rate under light exposure (continuous illumination at an intensity of 250 μmol·photons·m^−2^s^−1^) separately, as described in previous studies (60). The net photosynthetic oxygen evolution rate was determined from the oxygen evolution rate under light conditions together with the oxygen consumption rate measured in the dark.

### Measurement of whole-cell absorption spectra

Cells were collected during the exponential phase and the OD_730nm_ was adjusted to 0.3. Absorbance was measured using a SpectraMax M5 platform (Molecular Devices) over a wavelength range of 400 to 800 nm, with 1 nm intervals. Each curve represents the average of three replicates.

### Assay for PPSA activity

The reaction system was set up according to previous studies (86, 87), with a total volume of 100 μl containing 100 mM Tris-HCl [pH 8.0], 10 mM MgCl_2_, 10 mM ATP; 3 μg of enzyme and 5 mM pyruvate. The reaction was conducted in a 37 °C water bath for 5 min, initiated upon the addition of purified protein and terminated by the addition of 15 μl of 1% TFA. Specifically, a negative control group was included, which was conducted in the absence of enzyme supplementation. Pyruvate concentrations before and after the reaction were measured using the Pyruvate Assay Kit (Beyotime Institute of Biotechnology catalog no.: S0299S). The reaction catalyzed by PPSA is represented in the following equation. The reaction also requires the presence of divalent metal cations, particularly Mg^2+^ (37, 88):

$$Pyruvate+‑H_{2}O+ATP\overset{PPSA}{\to }Phosphoenolpyruvate+pi+AMP$$

### Data analysis

Each data was obtained from at least three independent biological replicates and is presented as the mean ± SD. Statistical significance was determined using two-tailed Student’s *t*-tests. ∗ represents *p* < 0.05, while ∗∗ represents *p* < 0.01.

## Data availability

The raw MS proteomics data and PRM data have been deposited in the public-access iprox database (http://www.iprox.org) with the identifier PXD072902.

## Supporting information

This article contains supporting information.

## Conflict of interest

The authors declare that they have no conflicts of interest with the contents of this article.
