# Supplementary material for: Lysine butyrylation modulates central carbon metabolism and photosynthesis in cyanobacteria
Source: J Biol Chem. 2026 Sep 17;302(11):113558. doi: 10.1016/j.jbc.2026.113558 (PMC13678012; doi:10.1016/j.jbc.2026.113558)
Supplement: Supplementary file 1 [file mmc1.pdf]

Supplementary Methods

The complete sequence of the pUltra-pylS/pylT plasmid used in this study (5′ to 3′) is provided in both text and graphical formats, with major genetic elements annotated. tac promoter is indicated by a dashed underline; MmPylS, the wild-type *M. mazei pylS* sequence, is indicated by a wavy underline. *rrnB* terminator, the terminator sequence of *E. coli rrnB*, is indicated by a double underline; MmPylT, the *M. mazei pylT* sequence, is indicated by underlining; *proK* promoter and *proK* terminator, the promoter and terminator sequence of *E. coli proK* respectively, are shown in bold. All other genetic elements are derived from the pUltra-CNF vector backbone. An annotated graphical representation of the complete plasmid sequence is also provided following the complete sequence.

CGACGCTCTCCCTTATGCGACTCCTGCATTAGGGAGCTGTTGACAATTAATCATC
GGCTCGTATAATGTGTGGAATTGTGAGCGGATAACAATTTCACAAAGGAGGTGCG
GCCGCATGGATAAAAAACCACTAAACACTCTGATATCTGCAACCGGGCTCTGGAT
GTCCAGGACCGGAACAATTCATAAAATAAAACACCACGAAGTCTCTCGAAGCAAA
ATCTATATTGAAATGGCATGCGGAGACCACCTTGTTGTAAACAACTCCAGGAGCA
GCAGGACTGCAAGAGCGCTCAGGCACCACAAATACAGGAAGACCTGCAAACGC
TGCAGGGTTTCGGATGAGGATCTCAATAAGTTCCTCACAAAGGCAAACGAAGAC
CAGACAAGCGTAAAAGTCAAGGTCGTTTCTGCCCCTACCAGAACGAAAAAGGCA
ATGCCAAAATCCGTTGCGAGAGCCCCGAAACCTCTTGAGAATACAGAAGCGGCA
CAGGCTCAACCTTCTGGATCTAAATTTTCACCTGCGATACCGGTTTCCACCCAAG
AGTCAGTTTCTGTCCCGGCATCTGTTTCAACATCAATATCAAGCATTTCTACAGGA
GCAACTGCATCCGCACTGGTAAAAGGGAATACGAACCCCATTACATCCATGTCTG
CCCCTGTTCAGGCAAGTGCCCCCGCACTTACGAAGAGCCAGACTGACAGGCTT
GAAGTCCTGTTAAACCCAAAAGATGAGATTTCCCTGAATTCCGGCAAGCCTTTCA
GGGAGCTTGAGTCCGAATTGCTCTCTCGCAGAAAAAAAGACCTGCAGCAGATCT
ACGCGGAAGAAAGGGAGAATTATCTGGGGAAACTCGAGCGTGAAATTACCAGGT
TCTTTGTGGACAGGGGTTTTCTGGAAATAAAATCCCCGATCCTGATCCCTCTTGA
GTATATCGAAAGGATGGGCATTGATAATGATACCGAACTTTCAAAACAGATCTTCA

GGGTTGACAAGAACTTCTGCCTGAGACCCATGCTTGCTCCAAACCTTTACAACTA
CCTGCGCAAGCTTGACAGGGCCCTGCCTGATCCAATAAAAATTTTTGAAATAGGC
CCATGCTACAGAAAAGAGTCCGACGGCAAAGAACACCTCGAAGAGTTTACCATG
CTGAACTTCTGCCAGATGGGATCGGGATGCACACGGGAAAATCTTGAAAGCATA
ATTACGGACTTCCTGAACCACCTGGGAATTGATTTCAAGATCGTAGGCGATTCCT
GCATGGTCTATGGGGATACCCTTGATGTAATGCACGGAGACCTGGAACTTTCCTC
TGCAGTAGTCGGACCCATACCGCTTGACCGGGAATGGGGTATTGATAAACCCTG
GATAGGGGCAGGTTTCGGGCTCGAACGCCTTCTAAAGGTTAAACACGACTTTAA
AAATATCAAGAGAGCTGCAAGGTCCGAGTCTTACTATAACGGGATTTCTACCAAC
CTGTAAGCGGCCGCGTTTAAACGGTCTCCAGCTTGGCTGTTTTGGCGGATGAGA
GAAGATTTTCAGCCTGATACAGATTAAATCAGAACGCAGAAGCGGTCTGATAAAA
CAGAATTTGCCTGGCGGCAGTAGCGCGGTGGTCCCACCTGACCCCATGCCGAA
CTCAGAAGTGAAACGCCGTAGCGCCGATGGTAGTGTGGGGTCTCCCCATGCGA
GAGTAGGGAACTGCCAGGCATCAAATAAAACGAAAGGCTCAGTCGAAAGACTGG
GCCTTGTTTGTGAGCTCCCGGTCATCAATCATCCCCATAATCCTTGTTAGCCTGC
AGGTAATTCCGCTTCGCAACATGTGAGCACCGGTTTATTGACTACCGGAAGCAGT
GTGACCGTGTGCTTCTCAAATGCCTGAGGCCAGTTTGCTCAGGCTCTCCCCGTG
GAGGTAATAATTGACGATATGATCAGTGCACGGCTAACTAAGCGGCCTGCTGACT
TTCTCGCCGATCAAAAGGCATTTTGCTATT**AAGGGATTGACGAGGGCGTATCTG**
CGCAGTAAGATGCGCCCCGCATTCGGAAACGTGATCATGTAGATCGAATGGACT
CTAAATCCGTTCAGTGGGGTTAGATTCCCCACGTTTCCGCCA**AATTCGAAAAGC**
CTGCTCAACGAGCAGGCTTTTTTGCATGCTCGAGCAGCTCAGGGTCGAATTTGC
CATGGCGGCCACCAGGTACCACCGGCGCCTCAGGCATTTGAGAAGCACACGGT
CACACTGCTTCCGGTAGTCAATAAACCGGTAAACCAGCAATAGACATAAGCGGCT
ATTTAACGACCCTGCCCTGAACCGACGACCGGGTCATCGTGGCCGGATCTTGC
GGCCCCTCGGCTTGAACGAATTGTTAGACATTATTTGCCGACTACCTTGGTGATC
TCGCCTTTCACGTAGTGGACAAATTCTTCCAACTGATCTGCGCGCGAGGCCAAG
CGATCTTCTTCTTGTCCAAGATAAGCCTGTCTAGCTTCAAGTATGACGGGCTGAT
ACTGGGCCGGCAGGCGCTCCATTGCCCAGTCGGCAGCGACATCCTTCGGCGC
GATTTTGCCGGTTACTGCGCTGTACCAAATGCGGGACAACGTAAGCACTACATTT

CGCTCATCGCCAGCCCAGTCGGGCGGCGAGTTCCATAGCGTTAAGGTTTCATTT
AGCGCCTCAAATAGATCCTGTTCAGGAACCGGATCAAAGAGTTCCTCCGCCGCT
GGACCTACCAAGGCAACGCTATGTTCTCTTGCTTTTGTCAGCAAGATAGCCAGAT
CAATGTCGATCGTGGCTGGCTCGAAGATACCTGCAAGAATGTCATTGCGCTGCC
ATTCTCCAAATTGCAGTTCGCGCTTAGCTGGATAACGCCACGGAATGATGTCGTC
GTGCACAACAATGGTGACTTCTACAGCGCGGAGAATCTCGCTCTCTCCAGGGGA
AGCCGAAGTTTCCAAAAGGTCGTTGATCAAAGCTCGCCGCGTTGTTTCATCAAG
CCTTACGGTCACCGTAACCAGCAAATCAATATCACTGTGTGGCTTCAGGCCGCC
ATCCACTGCGGAGCCGTACAAATGTACGGCCAGCAACGTCGGTTCGAGATGGC
GCTCGATGACGCCAACTACCTCTGATAGTTGAGTCGATACTTCGGCGATCACCG
CTTCCCTCATACTCTTCCTTTTTCAATATTATTGAAGCATTTATCAGGGTTATTGTCT
CATGAGCGGATACATATTTGAATGTATTTAGAAAAATAAACAAATAGCTAGCTCACT
CGGTCGCTACGCTCCGGGCGTGAGACTGCGGCGGGCGCTGCGGACACATACA
AAGTTACCCACAGATTCCGTGGATAAGCAGGGGACTAACATGTGAGGCAAAACA
GCAGGGCCGCGCCGGTGGCGTTTTTCCATAGGCTCCGCCCTCCTGCCAGAGTT
CACATAAACAGACGCTTTTCCGGTGCATCTGTGGGAGCCGTGAGGCTCAACCAT
GAATCTGACAGTACGGGCGAAACCCGACAGGACTTAAAGATCCCCACCGTTTCC
GGCGGGTCGCTCCCTCTTGCGCTCTCCTGTTCCGACCCTGCCGTTTACCGGATA
CCTGTTCCGCCTTTCTCCCTTACGGGAAGTGTGGCGCTTTCTCATAGCTCACAC
ACTGGTATCTCGGCTCGGTGTAGGTCGTTCGCTCCAAGCTGGGCTGTAAGCAAG
AACTCCCCGTTCAGCCCGACTGCTGCGCCTTATCCGGTAACTGTTCACTTGAGT
CCAACCCGGAAAAGCACGGTAAAACGCCACTGGCAGCAGCCATTGGTAACTGG
GAGTTCGCAGAGGATTTGTTTAGCTAAACACGCGGTTGCTCTTGAAGTGTGCGC
CAAAGTCCGGCTACACTGGAAGGACAGATTTGGTTGCTGTGCTCTGCGAAAGCC
AGTTACCACGGTTAAGCAGTTCCCCAACTGACTTAACCTTCGATCAAACCACCTC
CCCAGGTGGTTTTTTCGAAGGTTAAGTCAGTTGGGGAACTGCTTAACCTTCGATC
AAACCACCTCCCCAGGTGGTTTTTTCGTTTACAGGGCAAAAGATTACGCGCAGA
AAAAAAGGATCTCAAGAAGATCCTTTGATCTTTTCTACTGAACCGCTCTAGATTTC
AGTGCAATTTATCTCTTCAAATGTAGCACCTGAAGTCAGCCCCATACGATATAAGT
TGTAATTCTCATGTTAGTCATGCCCCGCGCCCACCGGAAGGAGCTGACTGGGTT

GAAGGCTCTCAAGGGCATCGGTCGAGATCCCGGTGCCTAATGAGTGAGCTAACT
TACATTAATTGCGTTGCGCTCACTGCCCGCTTTCCAGTCGGGAAACCTGTCGTG
CCAGCTGCATTAATGAATCGGCCAACGCGCGGGGAGAGGCGGTTTGCGTATTG
GGCGCCAGGGTGGTTTTTCTTTTCACCAGTGAGACGGGCAACAGCTGATTGCC
CTTCACCGCCTGGCCCTGAGAGAGTTGCAGCAAGCGGTCCACGCTGGTTTGCC
CCAGCAGGCGAAAATCCTGTTTGATGGTGGTTAACGGCGGGATATAACATGAGC
TGTCTTCGGTATCGTCGTATCCCACTACCGAGATGTCCGCACCAACGCGCAGCC
CGGACTCGGTAATGGCGCGCATTGCGCCCAGCGCCATCTGATCGTTGGCAACC
AGCATCGCAGTGGGAACGATGCCCTCATTCAGCATTTGCATGGTTTGTTGAAAAC
CGGACATGGCACTCCAGTCGCCTTCCCGTTCCGCTATCGGCTGAATTTGATTGC
GAGTGAGATATTTATGCCAGCCAGCCAGACGCAGACGCGCCGAGACAGAACTTA
ATGGGCCCGCTAACAGCGCGATTTGCTGGTGACCCAATGCGACCAGATGCTCCA
CGCCCAGTCGCGTACCGTCTTCATGGGAGAAAATAATACTGTTGATGGGTGTCT
GGTCAGAGACATCAAGAAATAACGCCGGAACATTAGTGCAGGCAGCTTCCACAG
CAATGGCATCCTGGTCATCCAGCGGATAGTTAATGATCAGCCCACTGACGCGTTG
CGCGAGAAGATTGTGCACCGCCGCTTTACAGGCTTCGACGCCGCTTCGTTCTAC
CATCGACACCACCACGCTGGCACCCAGTTGATCGGCGCGAGATTTAATCGCCGC
GACAATTTGCGACGGCGCGTGCAGGGCCAGACTGGAGGTGGCAACGCCAATCA
GCAACGACTGTTTGCCCGCCAGTTGTTGTGCCACGCGGTTGGGAATGTAATTCA
GCTCCGCCATCGCCGCTTCCACTTTTTCCCGCGTTTTCGCAGAAACGTGGCTGG
CCTGGTTCACCACGCGGGAAACGGTCTGATAAGAGACACCGGCATACTCTGCG
ACATCGTATAACGTTACTGGTTTCACATTCACCACCCTGAATTGACTCTCTTCCGG
GCGCTATCATGCCATACCGCGAAAGGTTTTGCGCCATTCGATGGTGTCCGGGAT
CT

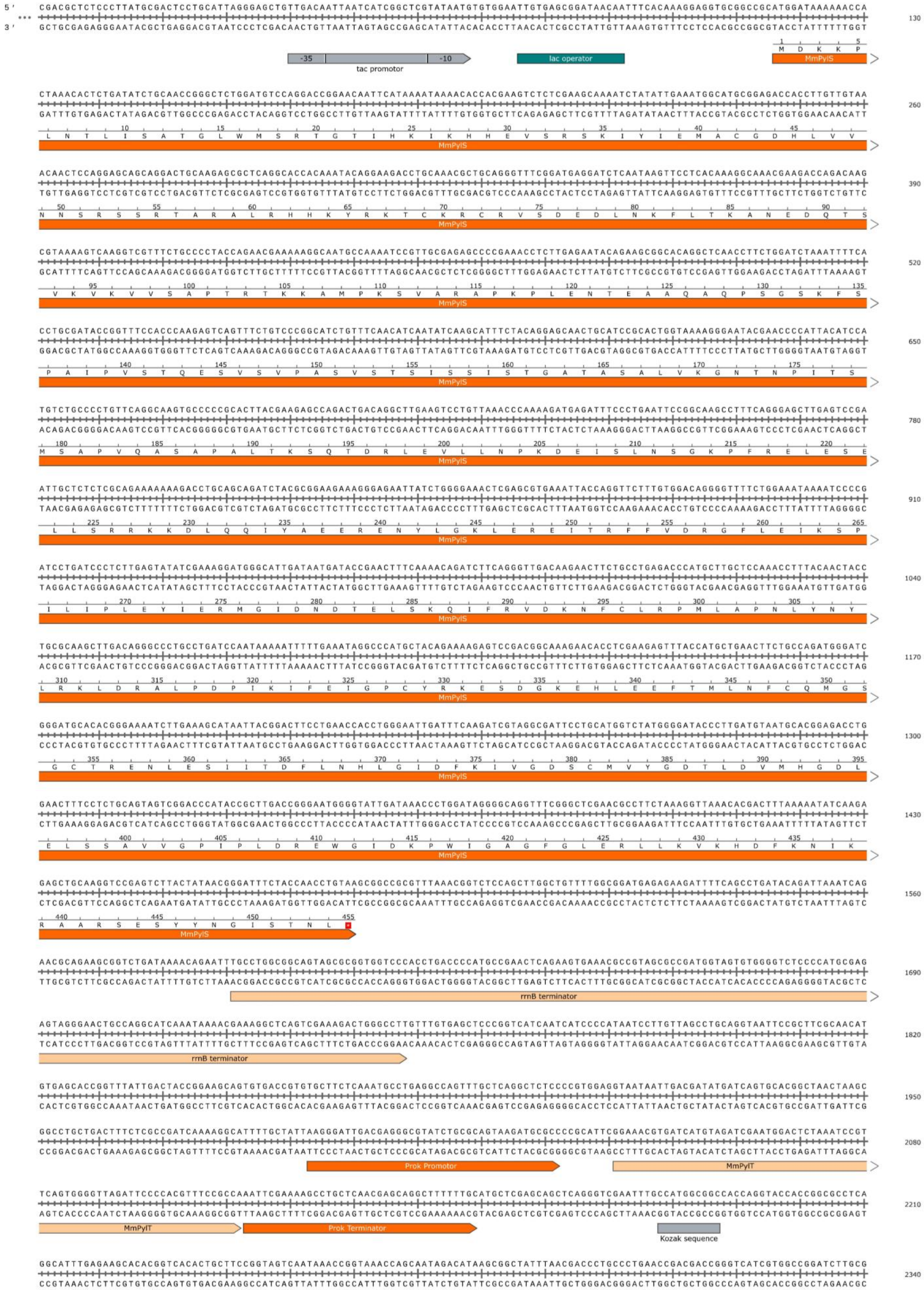

5'
3'
-35
-10
tac promotor
lac operator
MmPylS
rrnB terminator
Prok Promotor
MmPylT
Prok Terminator
Kozak sequence
130
260
390
520
650
780
910
1040
1170
1300
1430
1560
1690
1820
1950
2080
2210
2340

GCCCCTCGGCTTGAACGAATTGTTAGACATTATTTGCCGACTACCTTGGTGATCTCGCCTTTCACGTAGTGGACAAATTCTTCCAACTGATCTGCGCGCGAGGCCAAGCGATCTTCTTCTTGTCCAAGAT
CGGGGAGCCGAACTTGCTTAACAATCTGTAATAAACGGCTGATGGAACCACTAGAGCGGAAAGTGCATCACCTGTTTAAGAAGGTTGACTAGACGCGCGCTCCGGTTCGCTAGAAGAAGAACAGGTTCTA 2470

260 255 250 245 240 235
K G V V K T I E G K K V Y H V F E E L Q D A R S A L R D E E Q G L Y
SmR

AAGCCTGTCTAGCTTCAAGTATGACGGGCTGATACTGGGCCGGCAGGCGCTCCATTGCCCAGTCGGCAGCGACATCCTTCGGCGCGATTTTGCCGGTTACTGCGCTGTACCAAATGCGGGACAACGTAAG
TTCGGACAGATCGAAGTTCATACTGCCCGACTATGACCCGGCCGTCCGCGAGGTAACGGGTCAGCCGTCGCTGTAGGAAGCCGCGCTAAAACGGCCAATGACGCGACATGGTTTACGCCCTGTTGCATTC 2600

230 225 220 215 210 205 200 195 190
A Q R A E L I V P Q Y Q A P L R E M A W D A A V D K P A I K G T V A S Y W I R S L T L
SmR

CACTACATTTCGCTCATCGCCAGCCCAGTCGGGCGGCGAGTTCCATAGCGTTAAGGTTTCATTTAGCGCCTCAAATAGATCCTGTTCAGGAACCGGATCAAAGAGTTCCTCCGCCGCTGGACCTACCAAG
GTGATGTAAAGCGAGTAGCGGTCGGGTCAGCCCGCCGCTCAAGGTATCGCAATTCCAAAGTAAATCGCGGAGTTTATCTAGGACAAGTCCTTGGCCTAGTTTCTCAAGGAGGCGGCGACCTGGATGGTTC 2730

185 180 175 170 165 160 155 150 145
V V N R E D G A W D P P S N W L T L T E N L A E F L D Q E P V P D F L E E A A P G V L
SmR

GCAACGCTATGTTCTCTTGCTTTTGTCAGCAAGATAGCCAGATCAATGTCGATCGTGGCTGGCTCGAAGATACCTGCAAGAATGTCATTGCGCTGCCATTCTCCAAATTGCAGTTCGCGCTTAGCTGGAT
CGTTGCGATACAAGAGAACGAAAACAGTCGTTCTATCGGTCTAGTTACAGCTAGCACCGACCGAGCTTCTATGGACGTTCTTACAGTAACGCGACGGTAAGAGGTTTAACGTCAAGCGCGAATCGACCTA 2860

140 135 130 125 120 115 110 105
A V S H E R A K T L L I A L D I D I T A P E F I G A L I D N R Q W E G F Q L E R K A P Y
SmR

AACGCCACGGAATGATGTCGTCGTGCACAACAATGGTGACTTCTACAGCGCGGAGAATCTCGCTCTCTCCAGGGGAAGCCGAAGTTTCCAAAAGGTCGTTGATCAAAGCTCGCCGCGTTGTTTCATCAAG
TTGCGGTGCCTTACTACAGCAGCACGTGTTGTTACCACTGAAGATGTCGCGCCTCTTAGAGCGAGAGAGGTCCCCTTCGGCTTCAAAGGTTTTCCAGCAACTAGTTTCGAGCGGCGCAACAAAGTAGTTC 2990

100 95 90 85 80 75 70 65 60
R W P I I D D H V V I T V E V A R L I E S E G P S A S T E L L D N I L A R R T T E D L
SmR

CCTTACGGTCACCGTAACCAGCAAATCAATATCACTGTGTGGCTTCAGGCCGCCATCCACTGCGGAGCCGTACAAATGTACGGCCAGCAACGTCGGTTCGAGATGGCGCTCGATGACGCCAACTACCTCT
GGAATGCCAGTGGCATTGGTCGTTTAGTTATAGTGACACACCGAAGTCCGGCGGTAGGTGACGCCTCGGCATGTTTACATGCCGGTCGTTGCAGCCAAGCTCTACCGCGAGCTACTGCGGTTGATGGAGA 3120

55 50 45 40 35 30 25 20 15
R V T V T V L L D I D S H P K L G G D V A S G Y L H V A L L T P E L H R E I V G V V E
SmR

GATAGTTGAGTCGATACTTCGGCGATCACCGCTTCCCTCATACTCTTCCTTTTTCAATATTATTGAAGCATTTATCAGGGTTATTGTCTCATGAGCGGATACATATTTGAATGTATTTAGAAAAATAAAC
CTATCAACTCAGCTATGAAGCCGCTAGTGGCGAAGGGAGTATGAGAAGGAAAAAGTTATAATAACTTCGTAAATAGTCCCAATAACAGAGTACTCGCCTATGTATAAACTTACATAAATCTTTTTATTTG 3250

10 5 1
S L Q T S V E A I V A E R M
SmR
AmpR promoter

AAATAGCTAGCTCACTCGGTCGCTACGCTCCGGGCGTGAGACTGCGGCGGGCGCTGCGGACACATACAAAGTTACCCACAGATTCCGTGGATAAGCAGGGGACTAACATGTGAGGCAAAACAGCAGGGCC
TTTATCGATCGAGTGAGCCAGCGATGCGAGGCCCGCACTCTGACGCCGCCCGCGACGCCTGTGTATGTTTCAATGGGTGTCTAAGGCACCTATTCGTCCCCTGATTGTACACTCCGTTTTGTCGTCCCGG 3380

AmpR promoter
CDF-Ori

GCGCCGGTGGCGTTTTTCCATAGGCTCCGCCCTCCTGCCAGAGTTCACATAAACAGACGCTTTTCCGGTGCATCTGTGGGAGCCGTGAGGCTCAACCATGAATCTGACAGTACGGGCGAAACCCGACAGG
CGCGGCCACCGCAAAAAGGTATCCGAGGCGGGAGGACGGTCTCAAGTGTATTTGTCTGCGAAAAGGCCACGTAGACACCCTCGGCACTCCGAGTTGGTACTTAGACTGTCATGCCCGCTTTGGGCTGTCC 3510

CDF-Ori

ACTTAAAGATCCCCACCGTTTCCGGCGGGTCGCTCCCTCTTGCGCTCTCCTGTTCCGACCCTGCCGTTTACCGGATACCTGTTCCGCCTTTCTCCCTTACGGGAAGTGTGGCGCTTTCTCATAGCTCACA
TGAATTTCTAGGGGTGGCAAAGGCCGCCCAGCGAGGGAGAACGCGAGAGGACAAGGCTGGGACGGCAAATGGCCTATGGACAAGGCGGAAAGAGGGAATGCCCTTCACACCGCGAAAGAGTATCGAGTGT 3640

CDF-Ori

CACTGGTATCTCGGCTCGGTGTAGGTCGTTCGCTCCAAGCTGGGCTGTAAGCAAGAACTCCCCGTTCAGCCCGACTGCTGCGCCTTATCCGGTAACTGTTCACTTGAGTCCAACCCGGAAAAGCACGGTA
GTGACCATAGAGCCGAGCCACATCCAGCAAGCGAGGTTCGACCCGACATTCGTTCTTGAGGGGCAAGTCGGGCTGACGACGCGGAATAGGCCATTGACAAGTGAACTCAGGTTGGGCCTTTTCGTGCCAT 3770

CDF-Ori

AAACGCCACTGGCAGCAGCCATTGGTAACTGGGAGTTCGCAGAGGATTTGTTTAGCTAAACACGCGGTTGCTCTTGAAGTGTGCGCCAAAGTCCGGCTACACTGGAAGGACAGATTTGGTTGCTGTGCTC
TTTGCGGTGACCGTCGTCGGTAACCATTGACCCTCAAGCGTCTCCTAAACAAATCGATTTGTGCGCCAACGAGAACTTCACACGCGGTTTCAGGCCGATGTGACCTTCCTGTCTAAACCAACGACACGAG 3900

CDF-Ori

TGCGAAAGCCAGTTACCACGGTTAAGCAGTTCCCCAACTGACTTAACCTTCGATCAAACCACCTCCCCAGGTGGTTTTTTCGAAGGTTAAGTCAGTTGGGGAACTGCTTAACCTTCGATCAAACCACCTC
ACGCTTTCGGTCAATGGTGCCAATTCGTCAAGGGGTTGACTGAATTGGAAGCTAGTTTGGTGGAGGGGTCCACCAAAAAAGCTTCCAATTCAGTCAACCCCTTGACGAATTGGAAGCTAGTTTGGTGGAG 4030

CDF-Ori

CCCAGGTGGTTTTTTCGTTTACAGGGCAAAAGATTACGCGCAGAAAAAAAGGATCTCAAGAAGATCCTTTGATCTTTTCTACTGAACCGCTCTAGATTTCAGTGCAATTTATCTCTTCAAATGTAGCACC
GGGTCCACCAAAAAAGCAAATGTCCCGTTTTCTAATGCGCGTCTTTTTTTCCTAGAGTTCTTCTAGGAAACTAGAAAAGATGACTTGGCGAGATCTAAAGTCACGTTAAATAGAGAAGTTTACATCGTGG 4160

CDF-Ori

TGAAGTCAGCCCCATACGATATAAGTTGTAATTCTCATGTTAGTCATGCCCCGCGCCCACCGGAAGGAGCTGACTGGGTTGAAGGCTCTCAAGGGCATCGGTCGAGATCCCGGTGCCTAATGAGTGAGCT
ACTTCAGTCGGGGTATGCTATATTCAACATTAAGAGTACAATCAGTACGGGGCGCGGGTGGCCTTCCTCGACTGACCCAACTTCCGAGAGTTCCCGTAGCCAGCTCTAGGGCCACGGATTACTCACTCGA 4290

AACTTACATTAATTGCGTTGCGCTCACTGCCCGCTTTCCAGTCGGGAAACCTGTCGTGCCAGCTGCATTAATGAATCGGCCAACGCGCGGGGAGAGGCGGTTTGCGTATTGGGCGCCAGGGTGGTTTTTC
TTGAATGTAATTAACGCAACGCGAGTGACGGGCGAAAGGTCAGCCCTTTGGACAGCACGGTCGACGTAATTACTTAGCCGGTTGCGCGCCCCTCTCCGCCAAACGCATAACCCGCGGTCCCACCAAAAAG 4420

lacI

TTTTCACCAGTGAGACGGGCAACAGCTGATTGCCCTTCACCGCCTGGCCCTGAGAGAGTTGCAGCAAGCGGTCCACGCTGGTTTGCCCCAGCAGGCGAAAATCCTGTTTGATGGTGGTTAACGGCGGGAT
AAAAGTGGTCACTCTGCCCGTTGTCGACTAACGGGAAGTGGCGGACCGGGACTCTCTCAACGTCGTTCGCCAGGTGCGACCAAACGGGGTCGTCCGCTTTTAGGACAAACTACCACCAATTGCCGCCCTA 4550

lacI

ATAACATGAGCTGTCTTCGGTATCGTCGTATCCCACTACCGAGATGTCCGCACCAACGCGCAGCCCGGACTCGGTAATGGCGCGCATTGCGCCCAGCGCCATCTGATCGTTGGCAACCAGCATCGCAGTG
TATTGTACTCGACAGAAGCCATAGCAGCATAGGGTGATGGCTCTACAGGCGTGGTTGCGCGTCGGGCCTGAGCCATTACCGCGCGTAACGCGGGTCGCGGTAGACTAGCAACCGTTGGTCGTAGCGTCAC 4680

lacI

GGAACGATGCCCTCATTCAGCATTTGCATGGTTTGTTGAAAACCGGACATGGCACTCCAGTCGCCTTCCCGTTCCGCTATCGGCTGAATTTGATTGCGAGTGAGATATTTATGCCAGCCAGCCAGACGCA
CCTTGCTACGGGAGTAAGTCGTAAACGTACCAAACAACTTTTGGCCTGTACCGTGAGGTCAGCGGAAGGGCAAGGCGATAGCCGACTTAAACTAACGCTCACTCTATAAATACGGTCGGTCGGTCTGCGT 4810

lacI

GACGCGCCGAGACAGAACTTAATGGGCCCGCTAACAGCGCGATTTGCTGGTGACCCAATGCGACCAGATGCTCCACGCCCAGTCGCGTACCGTCTTCATGGGAGAAAATAATACTGTTGATGGGTGTCTG 4940
CTGCGCGGCTCTGTCTTGAATTACCCGGGCGATTGTCGCGCTAAACGACCACTGGGTTACGCTGGTCTACGAGGTGCGGGTCAGCGCATGGCAGAAGTACCCTCTTTTATTATGACAACTACCCACAGAC
lacI

GTCAGAGACATCAAGAAATAACGCCGGAACATTAGTGCAGGCAGCTTCCACAGCAATGGCATCCTGGTCATCCAGCGGATAGTTAATGATCAGCCCACTGACGCGTTGCGCGAGAAGATTGTGCACCGCC 5070
CAGTCTCTGTAGTTCTTTATTGCGGCCTTGTAATCACGTCCGTCGAAGGTGTCGTTACCGTAGGACCAGTAGGTCGCCTATCAATTACTAGTCGGGTGACTGCGCAACGCGCTCTTCTAACACGTGGCGG
lacI

GCTTTACAGGCTTCGACGCCGCTTCGTTCTACCATCGACACCACCACGCTGGCACCCAGTTGATCGGCGCGAGATTTAATCGCCGCGACAATTTGCGACGGCGCGTGCAGGGCCAGACTGGAGGTGGCAA 5200
CGAAATGTCCGAAGCTGCGGCGAAGCAAGATGGTAGCTGTGGTGGTGCGACCGTGGGTCAACTAGCCGCGCTCTAAATTAGCGGCGCTGTTAAACGCTGCCGCGCACGTCCCGGTCTGACCTCCACCGTT
lacI

CGCCAATCAGCAACGACTGTTTGCCCGCCAGTTGTTGTGCCACGCGGTTGGGAATGTAATTCAGCTCCGCCATCGCCGCTTCCACTTTTTCCCGCGTTTTCGCAGAAACGTGGCTGGCCTGGTTCACCAC 5330
GCGGTTAGTCGTTGCTGACAAACGGGCGGTCAACAACACGGTGCGCCAACCCTTACATTAAGTCGAGGCGGTAGCGGCGAAGGTGAAAAAGGGCGCAAAAGCGTCTTTGCACCGACCGGACCAAGTGGTG
lacI

GCGGGAAACGGTCTGATAAGAGACACCGGCATACTCTGCGACATCGTATAACGTTACTGGTTTCACATTCACCACCCTGAATTGACTCTCTTCCGGGCGCTATCATGCCATACCGCGAAAGGTTTTGCGC 5460
CGCCCTTTGCCAGACTATTCTCTGTGGCCGTATGAGACGCTGTAGCATATTGCAATGACCAAAGTGTAAGTGGTGGGACTTAACTGAGAGAAGGCCCGCGATAGTACGGTATGGCGCTTTCCAAAACGCG
lacI
lacI promoter

CATTCGATGGTGTCCGGGATCT 3′ 5482
GTAAGCTACCACAGGCCCTAGA 5′
lacI promoter
