# Supplementary material for: Lysine butyrylation modulates central carbon metabolism and photosynthesis in cyanobacteria
Source: J Biol Chem. 2026 Sep 17;302(11):113558. doi: 10.1016/j.jbc.2026.113558 (PMC13678012; doi:10.1016/j.jbc.2026.113558)
Supplement: Supplementary file 2 [file mmc13.docx]

**Supplementary Figures**

**Figure S1.** Distribution of butyrylated proteins based on the number of identified Kbusites per protein.

**Figure S2.** Sequence motifs for butyrylatedlysine residues in Syn7002.

**Figure S3.** Secondary structure prediction and surface accessibility of butyrylatedlysine residues in Syn7002.

**Figure S4.** GO classification of lysine butyrylated proteins in Syn7002 according to molecular function, cellular component and biological process.

**Figure S5.** Extracted ion chromatograms (XICs) of the K27-butyrylated ApcA peptide from individual biological replicates under low temperature (22°C), high temperature (47°C), high light (HL), nitrogen deprivation (−N), high salt (2.5 M NaCl), and normal control (NC) conditions.

**Figure S6.** Multiple sequence alignment of PPSA from various cyanobacterial species.

**Figure S7.** LC–MS/MS validation of WT and K143R variantsof PPSA. Representative MS/MS spectra of the peptide containing residue 143 from each sample are shown.

**Figure S8.** Construction and verification of Syn7002 mutant strains. (A) Construction of the Δ*ppsA* mutant strain. (B) Construction of the K143R mutant strain. (C) PCR verification of the Syn7002 mutant strains (Δ*ppsA* and K143R). (D) Sequencing verification of the K143R mutant strain.

**Figure S9.** Whole-cell absorption spectrum of wildtype (WT) and mutant strains (Δ*ppsA* and K143R) of Syn7002 under(A) normal condition (continuous illumination at 250 μmol·photons·m^−2^s^−1^) and (B) high lightcondition (continuous illumination at 2,000 μmol·photons·m^−2^s^−1^).Each curve represents the average of three replicates.

**Figure S10.** Non-photochemical quenching (NPQ) of WT and mutant strains (Δ*ppsA* and K143R) under (A) normal condition (continuous illumination at 250 μmol·photons·m^−2^s^−1^) and (B) high light condition (continuous illumination at 2,000 μmol·photons·m^−2^s^−1^).Data were obtained from three independent biological replicates, and are presented as the mean ± SD. Statistical significance was determined using two-tailed Student’s t-tests.

**Figure S11.** Crosstalk between lysine butyrylation and other lysine acylation modifications in Syn7002. (A) Overlap based on modified sites. (B) Overlap based on modified proteins.Khib, lysine 2-hydroxyisobutyrylation. Kac, lysine acetylation.Ksu, lysine succinylation.Kpr, lysinepropionylation.Kbu, lysine butyrylation.
